# Mobile application leads to psychological improvement and correlated neuroimmune function change in subjective cognitive decline

**DOI:** 10.1038/s41746-025-01765-1

**Published:** 2025-06-14

**Authors:** Merav Catalogna, Nira Saporta, Bar Nathansohn-Levi, Tal Tamir, Ariel Shahaf, Shira Molcho, Shai Erlich, Shahar Shelly, Amir Amedi

**Affiliations:** 1 The Baruch Ivcher Institute for Brain, Cognition, and Technology, Baruch Ivcher School of Psychology, Reichman University, Herzliya, Israel; 2 Remepy Health Ltd, Ramat Gan, Israel; 3 Department of Neurology, Rambam Medical Center, Haifa, Israel; 4 Rappaport Faculty of Medicine, Technion-Israel Institute of Technology, Haifa, Israel

**Keywords:** Neurodegeneration, Functional magnetic resonance imaging, Cytokines, Cognitive ageing, Emotion, Psychology, Therapeutics

## Abstract

Subjective cognitive decline (SCD) is a potential early marker of neurodegeneration, with negative affective states such as depression and anxiety significantly contributing to cognitive impairment. Digital treatments show promise, yet evidence of their use and efficacy in SCD remains limited. We studied 103 individuals aged 50-65, experiencing SCD and heightened anxiety, randomly assigned to a three-week mobile app program or waitlist control. Assessments included psychological measures, immunological analysis, and for a subgroup of the participants also resting-state functional connectivity (rsFC). The intervention significantly reduced proinflammatory mediators (TNF-α, IL-17, IL-23, MCP-1, IFN-γ, and IL-12) and improved depression, anxiety, resilience and well-being with sustained effect over a three-week follow-up. RsFC results show enhanced fronto-limbic connectivity correlated with the psychological and immunological changes, with the insula emerging as a key hub mediating these relationships. These findings highlight digital treatments as potential scalable, brain-immune targeted interventions for SCD and other medical conditions.

## Introduction

Subjective cognitive decline (SCD) is a prevalent form of cognitive impairment characterized by self-reported decline in thinking ability or memory^1,2^. While not all individuals with SCD develop a neurodegenerative condition, it is increasingly recognized as a potential early indicator of mild cognitive impairment (MCI) and Alzheimer’s disease (AD). Approximately 14.5% of individuals with SCD progress to dementia, and 26.6% to MCI within 5 years^3,4^. Furthermore, negative affective states, such as depression, anxiety, and stress, are associated with accelerated cognitive decline and progression to dementia^5,6^. Notably, SCD shows a stronger association with depressive symptoms than with objective measures of cognitive impairment^7^. Cognitive decline can negatively impact emotional well-being, creating a destructive reciprocal relationship between cognition and affect^8^. Therefore, addressing emotional health in individuals with SCD is highly important.

Depression, anxiety and stress have also been shown to profoundly impact the immune system^9,10^, primarily by inducing a pro-inflammatory state. This pro-inflammatory state has, in turn, a negative impact on the brain structure and function, potentially further exacerbating mood disorders^11,12^. This is particularly relevant to SCD, as growing research supports a strong theoretical basis for the reciprocal relationship between neurodegeneration and the immune system^13,14^. Additionally, research on mood disorders in degenerative diseases has indicated a significant association between psychological dysfunction and aberrant fronto-limbic circuit resting-state functional connectivity (rsFC)^15–17^. Specifically, depression and anxiety have been associated with atypical functional organization within the triple network model of brain connectivity, comprising the default mode network (DMN), the salience network (SN), and the central executive network (CEN)^18^. Within this triple-network framework the insula, a key node within the SN, plays a critical role in emotional awareness, interoception, and resilience by mediating dynamic switching between the DMN and CEN, thereby facilitating appropriate allocation of attentional and memory resources^19–21^. Additionally, disrupted rsFC within the fronto-limbic circuitry contributes to an imbalance between limbic structures, particularly the amygdala and hippocampus, and higher-order frontal regions, including the medial prefrontal cortex (mPFC), anterior cingulate cortex (ACC), and dorsolateral prefrontal cortex (dlPFC), which are integrally linked to the aforementioned networks^22–24^. These disruptions are thought to underlie key affective and cognitive symptoms observed in mood and anxiety disorders. Notably, alterations in rsFC, particularly in frontal regions, have been shown to be reversible with treatments such as antidepressants, and some of these changes have been proposed as predictors of treatment response^25,26^. With respect to brain-immune interactions, altered activity and connectivity in frontal brain areas, along with the amygdala and substantially with the insular cortex, have been implicated in the regulation of autonomic and neuroendocrine processes, particularly through bidirectional signaling mechanisms that influence systemic inflammation^27–29^. Consequently, in this study we focused not only on addressing the emotional health of people with SCD but also examined the influences on the immune system and functional connectivity related to any positive changes in their affective state.

While pharmacological management of negative affective states is common, their efficacy is limited, and older adults are often overprescribed these medications, despite the risk of drug-related side effects^30–32^. Given these limitations, there has been a rising interest in scalable non-pharmacological approaches. These approaches have been shown to bolster subjective memory performance and psychological well-being in SCD^33–35^, and they are also considered standard of care for affective disorders alongside medical treatment^36–40^. Additionally, there is an evolving body of literature pointing to the potential benefits of non-pharmacological interventions on immune system function^41,42^. Despite their effectiveness, non-pharmacological therapies such as cognitive behavioral therapy (CBT), acceptance and commitment therapy (ACT), mindfulness-based stress reduction (MBSR), mindfulness-based cognitive therapy (MBCT), and cognitive training remain challenging to access due to lack of trained providers, as well as geographic, financial, motivational and logistical barriers^43–45^. In an effort to standardize and scale such interventions, there has been expanding interest in digital treatments^46–50^; however, research on their use in older adults, particularly those with SCD, remains limited, and their underlying biological mechanisms are not well understood^41,42^. In the current study, we aimed to narrow these gaps by examining the psychological effects of a digital intervention for individuals with SCD, along with its potential biological and neuro-immune underlying mechanisms.

In a previous pilot study in individuals experiencing SCD, we demonstrated that daily digital intervention, combining psychological exercises with multisensory spatial navigation tasks, was associated with reduced depressive symptoms and lower saliva proinflammatory mediator levels. These changes were linked to a modified interplay between the DMN-SN rsFC^51^. In the current study, we aimed to explore the link between these three levels (psychological state, immune system function and brain rsFC) in a larger SCD cohort, using a controlled design and a more comprehensive serum analysis of immune mediators.

The RMPY-008 mobile application delivers a structured daily digitized treatment protocol, integrating top-down emotional regulation strategies, with bottom-up approaches that utilize cognitive multisensory modulation (a detailed description of the intervention protocol can be found in the Methods section and in Supplementary Table 1). Briefly, the intervention includes two main components: (1) psychological interventions adapted CBT, ACT, MBSR, MBCT, guided imagery, psychoeducation, and attention training; (2) a virtual spatial navigation exercises based on the principles of sensorimotor integration and visual masking (blindfold training, which has been demonstrated in prior studies to enhance neuroplasticity)^52,53^.

We assessed the impact of RMPY-008 on SCD participants who also report high levels of anxiety, in comparison to a waitlist-control group. The study comprised a three-week intervention with daily interactions. Participants were assessed before and after the intervention using psychological questionnaires and immunological serum analysis. To investigate possible neural substrates, resting-state fMRI was additionally performed in a subgroup of participants. We hypothesized that engagement with the RMPY-008 app would lead to significant improvements in psychological state and well-being. We also hypothesized that we would observe a positive influence on the immune system, through a reduction of pro-inflammatory cytokines. Exploring potential neural mechanisms and building on our prior findings with similar interventions, we hypothesized that the insula mediates the relationship between immune signaling and psychological processes in response to the intervention, given its role in brain-immune communication and its function as a hub of large-scale network activity. Furthermore, we tested if any psychological effect would be washed out, maintained, or further improved after an additional lower-intensity three-week follow-up period in the intervention group only. Finally, we evaluated adherence and satisfaction with RMPY-008, as these are critical factors for the success of any digital intervention.

The study demonstrated that RMPY-008 significantly reduced depression and situational anxiety while enhancing resilience and well-being, with improvements maintained or further enhanced during follow-up. Additionally, RMPY-008 was shown to significantly reduce peripheral pro-inflammatory cytokines, namely Tumor Necrosis Factor-alpha (TNF-α), Interleukin 17 (IL-17), Interleukin 23 (IL-23), Monocyte chemoattractant protein-1 (MCP-1), Interferon gamma (IFN- γ), and Interleukin 12 (IL-12). A key strength of our study was the identification of potential neural mechanisms underlying these effects, including changes in fronto-limbic functional connectivity, that correlated with both psychological and immunological changes, suggesting links between the brain, behavior, and the immune system. These findings suggest that RMPY-008 may modulate brain networks, leading to the promotion of both mental and immune system health in SCD.

## Results

### Participants

Of the 110 participants screened, 7 participants were excluded from analysis: two due to the loss of a close family member during the trial period (an exclusion criterion), one who found the application challenging to manage, and four who were lost to follow-up for personal reasons. Consequently, the study proceeded with 103 participants (Supplementary Fig. 1). In the test group, 51 participants agreed to take part in the follow-up period. Of these 3 participants fell ill and were unable to attend the last assessment, and 4 participants did not attend the final assessment without providing a specific reason (contact was lost). Therefore, 44 participants completed the follow-up assessment. The mean age of participants at inclusion was 55.8 ± 3.8 years (55.8 ± 4.2 in the control group, 55.8 ± 3.7 in the test group), and 72.8% were females (75% in the control group, 71.8% in the test group). The mean situational anxiety score in the STAIS-5 questionnaire was 13.2 ± 3.5 in the test group and 12.9 ± 3.8 in the control group. The baseline characteristics of these participants are detailed in Table 1. There were no significant differences between the control and test groups in these baseline characteristics.

**Table 1:** Baseline characteristics

Variable	Control	Trial	*p*-value
*N*	32	71	
Age (Years)	55.8 ± 4.2	55.8 ± 3.7	0.98
Gender (F)	75.0%	71.8%	0.92
Marital Status (married)	81.2%	66.2%	0.37
Marital Status (divorced)	9.4%	23.9%	
Marital Status (widower)	3.1%	2.8%	
Marital Status (single)	6.2%	7.0%	
Self-reporting anxiety or depression	46.9%	50.7%	0.88
BRCS	15.0 ± 2.4	14.6 ± 2.6	0.51
CES-D	26.4 ± 9.5	25.4 ± 9.8	0.62
MHC-SF	50.5 ± 17.2	50.2 ± 12.5	0.92
PSS-10	22.4 ± 4.9	21.5 ± 6.3	0.48
STAIS-5	13.2 ± 3.5	12.9 ± 3.8	0.72
STAIT-5	11.3 ± 2.6	11.4 ± 3.3	0.91
MoCA	25.3 ± 3.2	25.7 ± 3.2	0.64
Years of education	16.4 ± 2.0	16.4 ± 5.9	0.96

*BRCS* Brief Resilient Coping Scale, *CES-D* Center for Epidemiological Studies Depression, *MHC-SF* Mental Health Continuum Short Form, *PSS* Perceived Stress Scale, *STAIT-5* Trait Anxiety, *STAIS-5* State Anxiety, *MoCA* Montreal Cognitive Assessment, Group differences tested using *t*-test for continuous variables or Chi-Square for discrete variables, mean ± SD.

### The intervention effect on psychological well-being

**Fig. 1:**
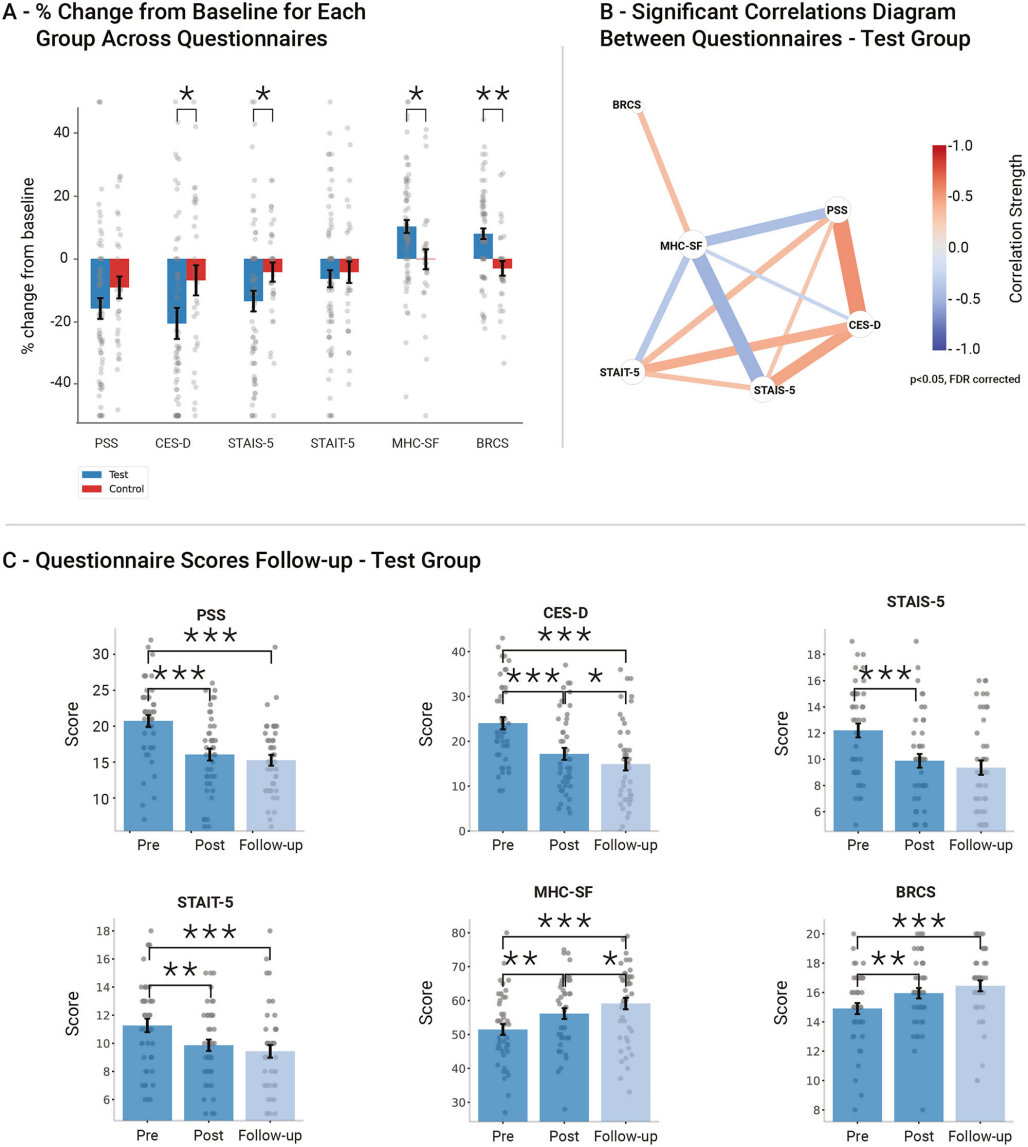
Psychological questionnaires results. **A** Percent change from pre-to-post intervention in each questionnaire, by group. Significant group-by-time interaction effects were found in the levels of CES-D (depression scale), STAIS-5 (situational anxiety), BRCS (resilience) and MHC-SF (well-being). Bars represent the mean; error bars represent the SEM. Extreme values were clipped at [-50,50] for visualization purpose only. A favorable result is represented by a reduction of scores in the CES-D, STAIS-5, STAIT-5 and PSS scales, and by an increase in scores in the BRCS and the MHC-SF scores. See supplementary Table 3 for details. **B** Correlation network diagram of psychological questionnaires in the Test Group. Nodes represent different questionnaires, with edges indicating significant correlations between individual changes from pre to post intervention. Edge thickness corresponds to the strength of the correlation, while edge color represents the correlation direction, ranging from blue (negative correlation, -1.0) to red (positive correlation, +1.0). Larger nodes indicate questionnaires with higher overall correlation strength. Edge locations maintain relative similarities among immune mediators using MDS (See methods). The dense network structure suggests that changes in psychological measures in the test group were systematic rather than incidental. **C** Questionnaire scores in the low-intensity follow-up Test group (*n* = 44), pre-intervention, post-intervention and after the low-intensity follow-up period. Bars represent the mean; error bars represent the SEM. Significant differences between measurements are indicated. See supplementary Table 5 for details. All p values are FDR corrected for multiple comparisons, *p < 0.05, **p < 0.01, ***p < 0.001.

To assess the psychological state, we utilized a range of standardized psychological questionnaires. All questionnaires showed acceptable reliability (Cronbach’s *α* > 0.7; Supplementary Table 2). We found a significant difference between groups in the change from baseline in CES-D (depression) [*β* = 1.77, SE = 0.77, *p* = 0.036], STAIS-5 (situational anxiety) [*β* = 0.75, SE = 0.31, *p* = 0.034], BRCS (resilience) [*β* = -0.73, SE = 0.2, *p* = 0.006] and MHC-SF (well-being) [*β* = -2.54, SE = 0.92, *p* = 0.021]. These results indicate a greater improvement in psychological scores in the test group relative to the controls (Fig. 1A; Supplementary Table 3). There was no significant interaction in PSS (perceived stress) and STAIT-5 (trait anxiety). Planned post-hoc analysis revealed significant improvements from baseline for all tested questionnaires in the test group, including the PSS and STAIT-5 questionnaires. However, no significant pre-post differences were found in the control group. This pattern indicates that improvements in psychological scores occurred within the test group, and not just in comparison to the control group.

To assess whether the changes in psychological state were correlated across questionnaires, we calculated pairwise correlations between the individual baseline-to-post intervention changes for each questionnaire. Figure 1B presents a correlation network diagram for the test group, displaying statistically significant correlations (*p* < 0.05, FDR corrected). In the test group, a well-connected network was observed: there were significant correlations among the changes in nearly all questionnaires, with the exception that changes in BRCS did not significantly correlate with changes in CES-D, PSS, STAIT-5, or STAIS-5. The direction of the correlation was positive among CES-D, PSS, STAIT-5, and STAIS-5, as well as between BRCS and MHC-SF. Conversely, MHC-SF showed negative correlation with CES-D, PSS, STAIT-5, STAIS-5. The consistent pattern of interrelated changes in the expected direction suggests that the observed psychological improvements in the test group were systematic rather than incidental. No significant correlations between questionnaires were observed in the control group (Supplementary Table 4), indicating that the changes in the control group may have been random or incidental. The stark contrast between the densely interconnected network in the test group and the fragmented structure in the control group suggests that the psychological changes in the test group reflect meaningful alterations in psychological state rather than chance fluctuations.

Next, we tested whether these psychological effects in the test group persisted or improved after a lower-intensity follow-up. Forty-four participants opted into the follow-up period and used the application for an additional 3 weeks at a reduced frequency (twice a week instead of daily). The positive effects were maintained in all psychological domains during this follow-up period. Specifically, for depression (CES-D, *p* = 0.026) and well-being (MHC-SF, *p* = 0.014) there were further significant improvements between the end of the 3 week intervention and the end of the 3 week follow-up (Fig. 1C, Supplementary Table 5).

### The intervention effect on pro-inflammatory cytokine levels

**Fig. 2:**
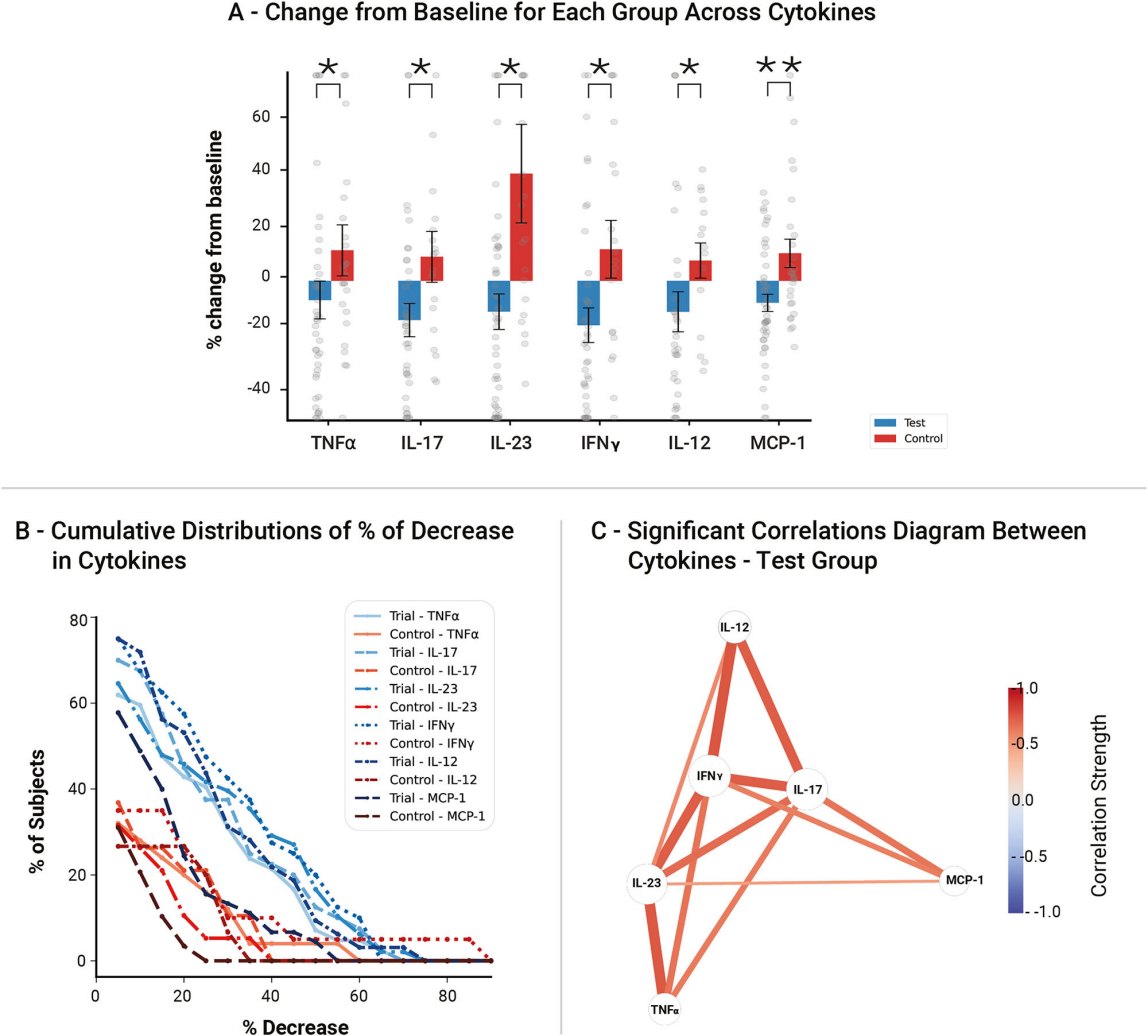
Immune mediators results. **A** Percent change from pre to post intervention in each cytokine, by group. Significant group-by-time interaction effects were found in the levels of TNF-α, IL-17, IL-23, MCP-1, IFN- γ, and IL-12. See supplementary Table 6 for details. **B** Cumulative distributions of the percentage of change in each cytokine level, the chart shows data only for participants for which there was a decrease greater than 5% in each specific mediator when comparing pre and post intervention levels (See supplementary Fig. 2 for details). There was a significant difference between the test and control groups across all cytokines - TNF-α, IL-17, IL-23, MCP-1, IFN- γ, and IL-12. **C** Correlation network diagram of immune mediators in the Test Group. Nodes represent individual cytokines, with edges indicating significant correlations between individual changes from pre to post intervention (*p* < 0.05, FDR corrected). Edge thickness corresponds to the strength of the correlation, while edge color represents the correlation direction, ranging from blue (negative correlation, -1.0) to red (positive correlation, +1.0). Larger nodes indicate cytokines with higher overall correlation strength. Edge locations maintain relative similarities among immune mediators using MDS (See methods). The dense network structure suggests strong interconnectivity among key immune mediators, particularly IL-23, TNFα, IFNγ, IL-17, and IL-12, indicating potential coordinated immune responses under test conditions. See supplementary Table 7 for details. (all *p*-values are FDR corrected for multiple comparisons, *p < 0.05, **p < 0.01).

When evaluating participants’ blood serum, we found significant group differences in the change from baseline for the following circulating immune mediators: TNF-α [β = -1.38, SE = 0.66, *p* = 0.039], IL-17 [β = -0.2, SE = 0.077, *p* = 0.023], IL-23 [β = -3.17, SE = 1.05, *p* = 0.011], MCP-1 [β = -128.99, SE = 38.59, *p* = 0.008], IFN- γ [β = -1.8, SE = 0.79, *p* = 0.035], and IL-12 [β = -0.84, SE = 0.37, *p* = 0.035] (Fig. 2A). These results signify a greater reduction in these immune mediators in the test group relative to the controls. Within-group post-hoc analysis of the immune system mediators, for which there was significant interaction, revealed significant improvement from baseline levels for all tested immune mediators in the test group, but no significant change from baseline in the control group (Supplementary Table 6). This indicates that absolute improvements in immune mediator levels occurred in the test group, not just related to the controls.

To further investigate the magnitude and the proportions of the reduction in these mediators within each group, we examined the subset of participants who showed >5% reduction in each mediator (Supplementary Fig. 2). Figure 2B shows the cumulative distributions of the percentage change in each mediator level. The test group consistently showed a higher degree of decrease. For example, 27% of the test group showed a reduction of 30% in IL-23, compared to only 4% of the control group showing such a reduction. To quantitatively compare the groups, we compared the full cumulative distributions of the changes. We found significant distribution differences for all these cytokines: TNF-α [U = 705, *p* = 0.024, *r* = 0.29], IL-17 [*U* = 537, *p* = 0.017, *r* = 0.33], IL-23 [*U* = 686, *p* = 0.008, *r* = 0.39], MCP-1 [*U* = 914, *p* = 0.012, *r* = 0.34], IFN- γ [*U* = 565, *p* = 0.017, *r* = 0.33], and IL-12 [*U* = 336, *p* = 0.029, *r* = 0.32].

To assess whether the changes in immune mediator levels were correlated, we calculated the pairwise correlations between the baseline-to-post changes for the cytokines that showed significant group interactions in the above analysis. Figure 2C presents a correlation network for the test group, showing statistically significant correlations (*p* < 0.05, FDR corrected). In the test group, a highly interconnected network was observed: reductions in nearly all cytokines were significantly correlated with each other. The only non-significant correlations were between TNF-α and IL-12 or MCP-1, and between IL-12 and MCP-1. This suggests that the changes in cytokine levels in the test group were systematic and functionally linked rather than incidental. In contrast, the control group exhibited a sparse network with few significant correlations, primarily limited to IL-23–TNFα, IL-17–MCP-1, and IL-17–IFNγ (Supplementary Fig. 3). The absence of widespread correlations in the control group suggests an uncoordinated immune response, reinforcing the notion that the coordinated cytokine changes in the test group were driven by the intervention rather than random variability (Supplementary Table 7 details the correlations).

### Seed-to-voxel based rsFC alterations within the fronto-limbic circuit

**Fig. 3:**
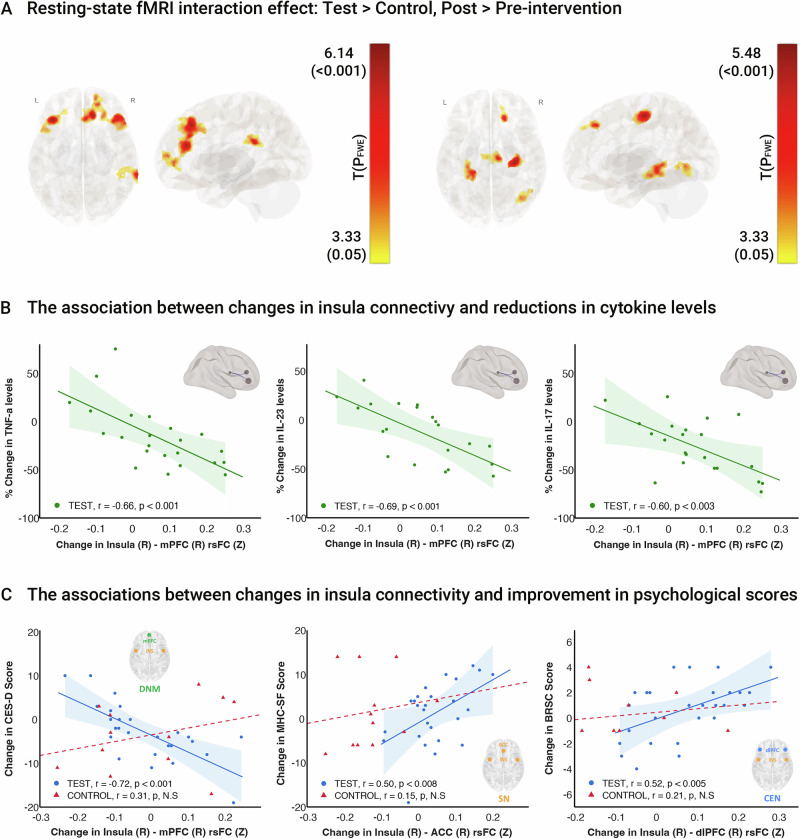
Association between brain plasticity, peripheral inflammation, and psychological state. **A** Seed-to-voxel group-by-time interaction ANOVA model maps of longitudinal group differences in the right and left insula seed and mPFC rsFC (voxel, *p* < 0.001, cluster, *p* < 0.05, FWE corrected, *n* = 27; test group, 12 control group). Insula is a structure that was recently suggested to play a key role in the brain-immune system axis in both humans and animal models (**B**). Significant association between increased right insula seed and mPFC rsFC and reduced IL-23, IL-17 and TNF-α pro-inflammatory cytokine levels (test group regression analysis, voxel *p* < 0.001, cluster *p* < 0.05, FDR corrected). **C** Significant association between alterations in right insula seed and mPFC, ACC, and dlPFC rsFC and improvement in depression, mental health, and resilience scores (regression analysis, voxel *p* < 0.001, cluster *p* < 0.05, FDR corrected, *n* = 27; test group, 12 control group). r, p Spearman rank correlation values, area, 95% prediction bounds, default mode network, DMN, salience network, SN, central executive network CEN. Brain images were generated using CONN (RRID:SCR_009550) https://web.conn-toolbox.org, v22a.

Seed-to-voxel based functional connectivity analysis revealed a significantly increased fronto-limbic rsFC in the test group compared to the control group, as depicted in Fig. 3A and Supplementary Table 8. The most prominent group-by-time interactions were observed in connectivity between the right insula rsFC with several regions: the left and right dorso-lateral PFC (dlPFC) (*T* = 5.44, 5.92; *K* = 474, 336; *p* < 0.001 FWE corrected respectively), the right dorsal anterior cingulate cortex (dACC) (*T* = 6.14; *K* = 403; *p* < 0.001 FWE corrected) and the right medial prefrontal cortex (mPFC) (*T* = 5.05, *K* = 165, *p* < 0.002 FWE corrected). Significant group-by-time interactions were also observed in the left insula rsFC with the left hippocampus (*T* = 4.63; *K* = 286; *p* < 0.001 FWE corrected), and with the right precentral gyrus (*T* = 5.48; *K* = 266; *p* < 0.001 FWE corrected). These regions overlap with the default mode network (DMN), central executive network (CEN), and salience network (SN), coordinating the modulation of immune and cognitive function, as well as emotional states^54,55^. Additional decreased significant group-by-time effects were also observed in the right amygdala rsFC with the left middle frontal gyrus, Broca’s area 44, (*T* = -5.78; *K* = 219; *p* < 0.001 FWE corrected), possibly related to top-down control over the amygdala, due to tasks involving emotion regulation.

Testing the amygdala and hippocampus seeds, group-by-time alterations were not observed, however in the test group increased connectivity was demonstrated between the left amygdala and the right mPFC (*T* = 5.25; *K* = 414; *p* < 0.001 FWE corrected, Supplementary Fig. 4A), and decreased connectivity between the right amygdala and the left precuneus (*T* = -5.27; *K* = 377; *p* < 0.001 FWE corrected, Supplementary Fig. 4B). Increased connectivity was also demonstrated between the left and right hippocampus and the ACC (*T* = 4.11; *K* = 183; and *T* = 4.52; *K* = 67, respectively, *p* < 0.05 FDR corrected, and Supplementary Fig. 4C, D) in the test group.

### Association between immune system function, psychological state, and brain rsFC

A key strength of our approach is that it allowed us to investigate the relationship between cytokine level changes, psychological state, and brain connectivity alterations using voxel-based regression analysis (*p* < 0.05 FDR corrected; Results are highlighted in Fig. 3B). Due to the small sample size of the control MRI subset and insufficient detectable cytokine data (above the lower limit of detection) in that subset, this particular analysis was limited to the test group.

With a focus on brain regions exhibiting significant group-by-time connectivity differences, we observed significant negative correlations between increased right insula-mPFC rsFC and the extent of reductions in IL-17, IL-23 and TNF-α levels (Fig. 3B). Additionally, increases in right insula-ACC rsFC were associated with greater reductions of IL-23 and TNF-α levels. Further correlations within the insular network were found between the right insula-hippocampus rsFC and reductions in IL-17, IL-23, TNF-α, MCP-1, IL-12 and IFN- γ levels, and between the left insula-orbitofrontal cortex (OFC) and the paras-orbitalis (PORB) rsFC and reductions in il-17, IL-23, IFN- γ and TNF-α levels. These widespread associations related to limbic structures strengthen the hypothesis that the insula serves as key integrator of brain-immune interactions in the context of psychological processes. Lastly, reductions in IL-17, IL-23, IFN- γ MCP-1 and IL-12 were correlated with increases in amygdala-mPFC rsFC. All significant correlations are listed in Supplementary Table 9.

We further explored the brain-psychological state associations and found significant negative correlations between the extent of reductions in CES-D score and the increase in rsFC between the right insula and the right mPFC (*r* = -0.72, *p* < 0.001; Compared to control group, control, N.S, Fig. 3C). Additionally, positive correlations were found between the rsFC of the right insula with the right ACC and extent of improvements in MHC-SF scores, as well as between increased right insula with the right dlPFC rsFC and improvements in BRSC scores (*r* = 0.50, *p* < 0.008; *r* = 0.52, *p* < 0.005 respectively, control, N.S; Fig. 3C). These findings demonstrate the role of the insula in modulating activity across the triple network SN, DMN, and CEN connectivity.

### Participants’ adherence and satisfaction

**Fig. 4:**
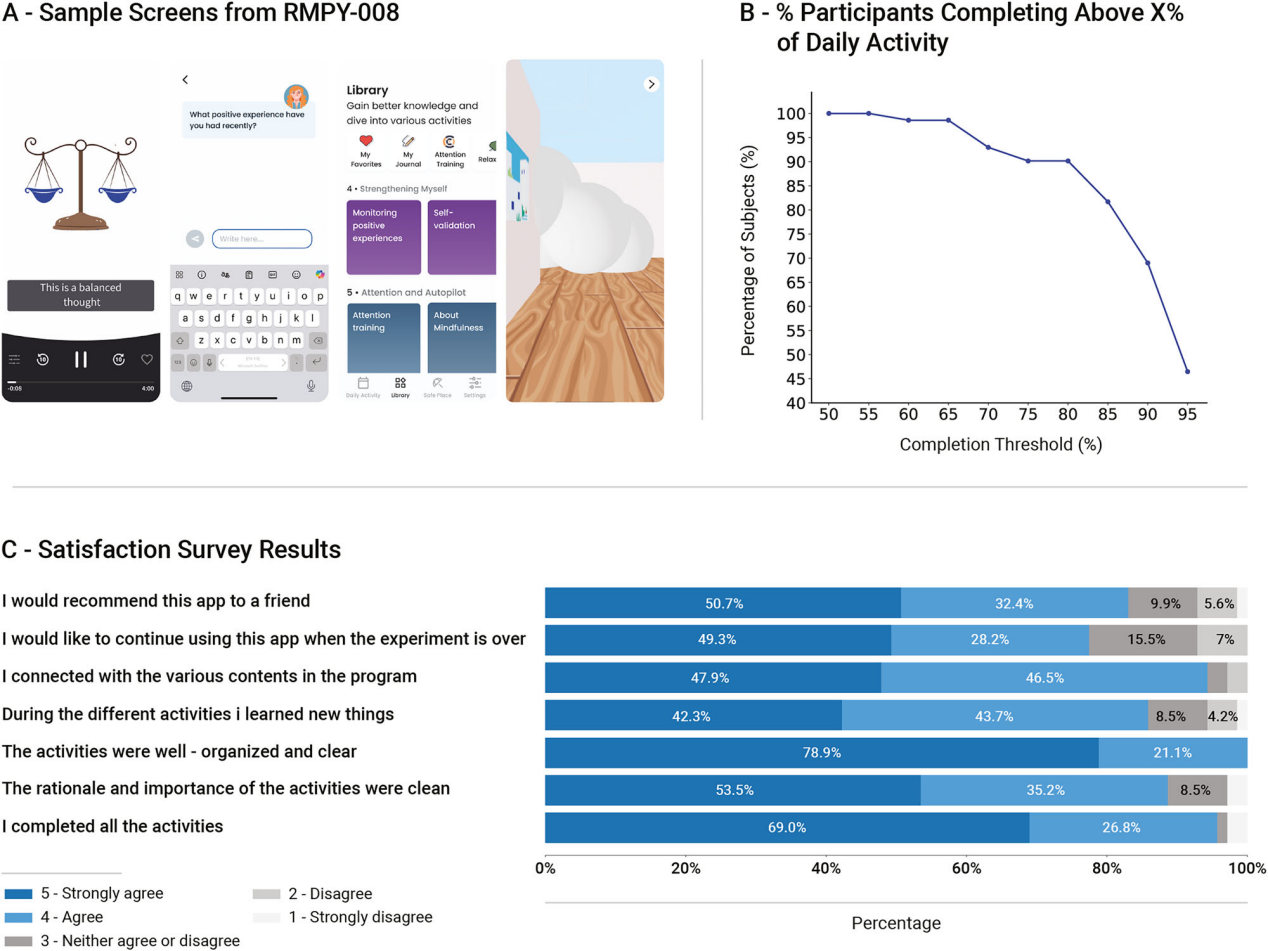
Adherence and satisfaction from the app. **A** Sample screens from the application (from left to right) – psychological intervention – video, psychological intervention – interactive activity, content library, partially visual-masked maze. **B** Accumulation curve illustrating the cumulative percentage of subjects completing various percentages of the program daily activities. **C** Results of satisfaction survey, percentage of test group participants responding 5 (strongly agree), 4 (agree), 3 (neither agree or disagree), 2, (disagree) and 1 (strongly disagree) are shown for items from the survey. For improved readability, percentage is shown for response options that received >3% of the responses. See Supplementary Table 10 for details.

At the end of the study, participants provided feedback on the RMPY-008 app (see sample screens in Fig. 4A). High engagement was observed, with participants actively using the RMPY-008 app an average of 19.8 days out of the 21 days of the experiment period (94.3% adherence). Figure 4B shows the accumulation curve illustrating the cumulative percentage of subjects completing various percentages of the program. High adherence and interest were evident: 90.14% of the test group participants completed at least 80% of the entire 3 week program. Participants rated their satisfaction with the app across several categories. Participants reported strong adherence, with 95.8% agreeing they completed all activities (69.0% strongly agreed). Content engagement was high: 94.4% felt connected to the material, 86.0% learning new things, and 77.5% expressed a desire to continue using the app after the study (see Fig. 4C and Supplementary Table 10).

## Discussion

In this study, we investigated a mobile digital treatment program designed to improve psychological function in individuals with SCD. Participants engaged in a 3 week daily protocol, combining evidence-based psychological interventions with cognitive training that utilizes multisensory modulation. We found significant improvement in mood and mental health manifested by reduced levels of depression and situational anxiety levels along with enhanced resilience and overall well-being (Fig. 1, Supplementary Table 3). Measuring immune mediators in blood serum before and after app use, we detected a significant decrease in peripheral pro-inflammatory cytokines, including TNF-α, IL-17, IL-23, MCP-1, IFN-γ, and IL-12 (Fig. 2, Supplementary Table 6). Using rsFC scans and voxel-based regression analysis, we found that psychological and immunological changes were associated with altered connectivity of the insula, alongside higher-order frontal DMN, SN and CEN regions, supporting its hypothesized role (Fig. 3, Supplementary Tables 8-9). These findings reinforce the insula’s function as a key hub in brain-immune communication, linking immune signaling with psychological processes in response to the intervention. The observed changes in insular connectivity may suggest its involvement in bidirectional interactions between the brain and immune system^55^, promoting positive behavioral changes and enhancing top-down regulation of immune function. Finally, participants reported high satisfaction with the app and demonstrated strong adherence, indicating the feasibility of integrating this program into daily routines (Fig. 4, Supplementary Table 10). Targeting psychological well-being in older adults with SCD may support objective cognitive functioning by reducing stress, inflammation, enhancing emotional resilience, and modulating brain-immune interactions. These effects may help preserve neural networks involved in memory and executive function, providing a potential strategy against progressive cognitive decline. Therefore, the inclusion of both psychological and cognitive treatment components reflects their complementary and interactive roles, as emotional regulation and cognitive engagement jointly influence neural plasticity and inflammation. These results suggest that the RMPY-008 mobile app has the potential to promote mental health, improve brain function and modulate immune system health in individuals with SCD. Future studies should further explore its efficacy.

The digital treatment led to significantly reduced depression and situational anxiety levels, and a significant increase in resilience and well-being, in the test group compared to the control group. Moreover, in the test group most of these psychological changes were significantly inter-correlated in the expected direction, whereas such correlations were absent in the control group, further attesting to the robustness of the finding. This positive psychological change aligns with prior research showing that daily digital interventions, such as mindfulness^56,57^ and CBT^58^, can be effective. Specifically, the RMPY-008 app used a combination of evidence-based approaches, which has been shown to promote greater change than using a single approach alone, if integrated thoughtfully^59,60^. The application of sensory integration, sensory substitution and deprivation principles (see Methods) may have contributed to the substantial effect observed in a relatively short time, as these approaches have been demonstrated to accelerate learning and induce sensory and cognitive network balance in past studies^52,53,61^. This combination aligns with the interest in leveraging not only psychological interventions, but also novel neuroscience-based approaches in digital treatments for older adults’ cognitive and mood disorders^62^. Adherence to and acceptability of the mobile program were high, possibly due to the focus on making the digital interventions short and interactive, an approach that is suggested to be associated with better outcomes^63^. Importantly, the psychological improvements were sustained over a 3 week low-intensity follow-up period, demonstrating that the treatment may also be beneficial even when administered in a non-daily fashion. These results demonstrate the positive potential of RMPY-008 as a digital treatment for improving emotional well-being in SCD.

Following the digital treatment, we observed significant reductions in several pro-inflammatory immune mediators in the test group compared to the control group, namely in the levels of TNF-α, IL-17, IL-23, IFN- γ, IL-12 and MCP-1. Moreover, in the test group many of the changes in these immune mediators were significantly correlated with each other (whereas very few correlations were found in the control group), further supporting the coordinated nature of the immune response to the intervention. Modulating these specific immune mediators is highly relevant to the psychological improvements observed, as depression has been associated with peripheral over-activation of the IL-23/IL-17 pathway^64^, and increased levels of TNF-α and IL-12^65,66^. Our findings are in agreement with other reports showing that non-digital interventions based on mindfulness and CBT can positively influence the immune system^41,42,67^. Specifically, mindfulness-based stress reduction led to decreased IL-17 and IL-23 levels in patients with autoimmune hepatitis^68^, as well as levels of TNF-α in patients with anxiety^69^. Cognitive behavioral therapy and psychodynamic therapy have been shown to reduce TNF-α in depressed patients, and cognitive behavioral stress management led to reduction in IL-12 levels in multiple sclerosis patients^70–72^. Potentially, the association between inflammation and mood disorders may be mediated by chronic stress-related activation of the hypothalamic-pituitary-adrenal (HPA) axis. Sustained HPA-axis activation leads to elevated stress hormone levels^73,74^. This hormonal imbalance was shown to affect immune cell function and shift the cytokine profile towards a pro-inflammatory state^75,76^. Notably, the reductions in IFN-γ and IL-12 in our study may not be clearly beneficial. While IFN-γ is typically viewed as pro-inflammatory and can drive neuroinflammation^77^, some studies suggest that greater IL-12/IFN-γ axis activation may be protective against cognitive decline and early-stage AD progression^78–80^. Therefore, further research is required to better interpret this finding. The observed reduction in pro-inflammatory immune mediators suggests that the mobile digital treatment may have reduced inflammation, possibly through its positive influence on their psychological well-being. This finding strengthens the body of knowledge that suggests that positive modulation of the immune system can be the result of non-pharmacological treatment, and specifically in digital form.

We observed a post-intervention increase in rsFC between the right insula and frontal regions, specifically the left and right dlPFC, right dACC and right mPFC in the test group compared to controls. Notably, these frontal regions are key nodes within three major cognitive networks: the dlPFC within the CEN, the dACC within the SN, and the mPFC within the DMN^54^. Prior studies have demonstrated reduced rsFC between the anterior insula and frontal regions in individuals with subthreshold depression^24,81,82^ and drug-naïve depressed patients^83–85^, suggesting a network-level dysregulation in these populations. Our findings may reflect the enhancement of this connectivity pattern. This interpretation aligns with the model proposed by Menon and Uddin^21^, in which the insula serves as a critical hub within the SN, facilitating dynamic switching between the DMN and CEN. The observed increase in insula–frontal connectivity may therefore indicate enhanced network coordination, contributing to improved mood regulation. This is further supported by the significant correlation between increased rsFC between the insula with all three frontal regions and improvements in depression, well-being and resilience scores observed in our study following the intervention.

The ACC and mPFC, as a part of the SN and DMN, have been specifically implicated in regulating autonomic and neuroendocrine processes that relate to systemic inflammation via bidirectional signaling mechanisms^27,28^. Additionally, neuronal activation within the amygdala and the insular cortex is related to peripheral inflammatory stimuli^86^, and bidirectional functional loops exist between the insula and the immune system^29,87^. In our study, increased rsFC between the insular and frontal regions as well as between the amygdala and frontal regions was associated with reduced inflammation in the IL17/ IL-23 immune axis, suggesting that enhanced connectivity between these regions may play a role in modulating inflammatory responses. Taken together, this comprehensive controlled study provides direct evidence of correlations between the influences of the digital treatment on all three levels (psychological, immunological, and neural). Beyond reinforcing the psychological and immunological findings of this specific study by suggesting a potential mediating neural mechanism, these results also offer a deeper understanding of the neuroimmunology basis of digital non-pharmacological treatments in general.

Some study limitations of the study should be considered when interpreting our findings. While acknowledging these limitations is crucial, it is important to consider them in light of the complex design and innovative nature of this digital treatment study, which encompassed the concurrent use of behavioral, neural, and immune system outcomes in the same individuals.

A notable limitation of the study is the use of a waitlist control group instead of a placebo control group. In general, although waitlist control designs are commonly used, trials that employ them tend to report larger effect sizes^88^. Creating a convincing placebo for a digital treatment is particularly challenging, as it is difficult to develop a “sham” digital intervention that lacks all major active components^89,90^. That said, the results of this study can help inform the creation of such a sham application, and future studies should ideally use a double-blind, sham-app controlled design. Another limitation of the study is the relatively small sample size, particularly of the control group, which rendered a few analyses (specifically the combined MRI and immune analyses) not statistically viable. More specifically, unbalanced sample sizes across groups may have impacted statistical power, thereby affecting the detection of significant results in a smaller sub-group. This limitation should be considered when interpreting between-group differences. While the multitude of consistent results attests to their reliability, future studies should increase the sample size to allow for a more robust statistical analysis. In addition, the study was relatively short. Although there were some indications of prolonged effects (e.g., in the follow-up group results), longer follow-up is required in future studies to assess the stability of the observed effects. An additional limitation is that we recruited participants with subjective cognitive complaints and self-reported higher anxiety, yet these individuals were otherwise healthy and without a specific medical or psychological diagnosis. This also means that participants’ baseline immune function and brain connectivity were relatively normal. While SCD is considered a potential early indicator of dementia, this recruitment approach limits the generalizability of the findings to different patient populations. Additional studies are needed to determine whether this type of intervention can benefit patient populations, and whether the neuroimmune changes we observed apply to those groups as well.

Another limitation is that we did not use memory or objective cognitive function assessments. This decision was due to our focus on the psychological and immune effects, as well as the relatively short trial period. However, assessing both subjective and objective cognitive function before and after the intervention would be beneficial to establish a link between improvements in psychological and immune measures, and the core cognitive complaints of SCD. Additionally, the study design does not enable disentangle the specific contributions of the psychological and navigation components. Future studies should include component-specific arms to clarify their respective effects. The study focused on a specific age group (50lrig65). While this is a relevant age group for the investigation of SCD, (about 20% of people ≤65 report SCD^2^), future studies should include older adult populations. This is particularly relevant when looking at SCD as a potential early indicator of MCI and AD, with the age of 60 being suggested as the cutoff for “SCD plus” in studies of prodromal AD that are unrelated to genetic mutations^91^. Additionally, the study was conducted in a single location, and future studies should include diverse geographies and populations to enhance cultural applicability. Finally, the use of smart phones has become ubiquitous, including among older adult population^92,93^, supporting the feasibility of a mobile-based approach. However, in the current study the participants were recruited using social media, which may have created a selection bias towards a more technology savvy subgroup. In future larger studies, we plan to explore alternative recruitment strategies to assess feasibility across more diverse populations.

The observed reduction in pro-inflammatory cytokines has potential relevance not only to the participants’ psychological state but also directly to neurodegeneration. Neuroinflammation and systemic inflammation have been identified to contribute to the development of neurodegenerative diseases and age-related cognitive impairments such as MCI and AD^94–96^. Specifically, evidence links MCP-1^97^, TNF-α^98^, IL-23^99^, and IL-17^100^ to these processes. Additionally, studies have highlighted the significance of the immune system for the lifelong maintenance of the brain^101^. Future research could examine the relevance of such a digital treatment also in more pathological neurodegeneration.

The study’s favorable outcomes align with advancements in digital medicine. In particular, it has been suggested that artificial intelligence (AI) could enhance digital treatments by leveraging personal variables to predict and optimize individualized treatment plans^102,103^. To increase long term adherence, among other core needs expressed by patients in this study, our next application will aim to include AI-driven features to increase user-treatment engagement, including an “AI Care Coordinator” to provide scalable support for app navigation and treatment guidance, AI-based personalization of the therapeutic program, and conversational AI tools to help users practice important psychological skills. Such adaptations could possibly offer standardized, high quality integrative care to broader populations, making this a promising direction for future studies.

Finally, in recent years, the U.S. Food and Drug Administration cleared prescription digital treatments for several conditions, including sleep disorders, depression, ADHD, and anxiety^46^. Investigating the underlying biological mechanisms of digital treatments is crucial, especially as these interventions become more widely used as prescription therapies and may even become part of combined digital–drug treatment regimens in the future^104^. The ability to positively influence the psychological state using a standardized mobile digital treatment protocol could be relevant in many medical conditions, such as cancer, cardiovascular, neurological, metabolic and autoimmune diseases, as it affects adherence to medication and self-care, prognosis, morbidity, and mortality. Moreover, chronic inflammation is integral to the pathogenesis of many prevalent chronic conditions, and combining anti-inflammatory drugs with treatment-as-usual has shown benefits in conditions, such as Parkinson’s disease^105^, depression^106^ and cancer^107^. This suggests that using an app like RMPY-008 to modulate inflammatory responses could enhance the efficacy of certain medications, beyond the benefits achieved by improving a patient’s mental state. Future studies should explore whether digital apps can improve medical outcomes when used in hybrid with pharmacotherapy.

This study demonstrated the effectiveness of RMPY-008, a novel mobile application designed to target psychological state and immune function in individuals reporting SCD and elevated anxiety. The intervention led to significant reductions in depression and anxiety, along with improvements in resilience and overall well-being. Additionally, reduced levels of inflammatory mediators were observed, correlating with positive changes in fronto-limbic network connectivity, particularly between the insula, amygdala, and frontal DMN regions. These findings suggest that RMPY-008’s effect may involve bidirectional interactions between brain and immune function achieved by combining both top-down emotional regulation strategies with bottom-up multisensory modulation approaches. In addition, the high adherence and acceptance observed further highlight the feasibility of digital interventions in SCD population. The pioneering use of behavioral, neural, and immune measurements in the same individuals pre- and post-intervention was instrumental in promoting our understanding of the underlying mechanisms of action of the digital treatment. This methodology, along with the positive study results, has potential implications for the development of digital treatments as standalone or hybrid digital-drug interventions for SCD and other medical conditions.

## Methods

### Study design and participants

The study was undertaken at the Baruch Ivcher Institute for Brain, Cognition & Technology (BCT) within the School of Psychology at Reichman University, Israel. The study had a waitlist-controlled design and recruited 110 healthy adults aged between 50 and 65, with a subjective report of cognitive decline (SCD) and increased situational anxiety, as reflected in a STAIS-5 score of ≥10^108^. In accordance with the conceptual framework for research in SCD, offered by Jessen et al.^91,109^, participants were determined to have SCD by providing a self-report on decreased cognitive function compared to the past. Participants were recruited through social media. Participants were excluded if they had a recent loss of a first degree relative, a history of malignancy, brain injuries or surgery, neurodegenerative or psychiatric disorders, or recent involvement in mindfulness or meditation practices (see Supplementary Information for further details). An MRI sub-study was a pre-planned component of the study design. Although MRI examinations were offered to all eligible participants, only a subset underwent imaging due to participant withdrawals (e.g., claustrophobia, scheduling constraints), exclusions based on standard contraindications (e.g., presence of metal implants, age >65 years due to the non-medical facility setting), and budgetary limitations associated with the feasibility-focused nature of this initial phase.

At screening, participants e-signed consent for the screening process and completed online screening questionnaires. The study staff contacted potential study participants by phone to complete missing or ambiguous data. The Principal Investigator then reviewed the screening data and enrolled eligible participants. Participants were then randomly assigned to a treatment or control group, in a 2:1 ratio.

During the baseline assessment, participants signed an informed consent form and completed the MOCA evaluation and the psychological questionnaires, followed by the collection of whole blood samples in EDTA collection tubes by a registered nurse. They then underwent an fMRI scan, and the RMPY-008 app was installed on the test group participants’ smartphones and configured to remind the participants daily to use the app. After 21 days, participants returned to the site for the post-intervention assessment, completed the psychological questionnaires, and underwent an fMRI scan, followed by collection of a whole blood sample in EDTA collection tubes by a registered nurse. The test group was also asked to complete a user experience questionnaire to provide feedback on the app’s features and usability, as well as their level of satisfaction. As an option, and subjected to providing additional consent, test group participants were offered to participate in a follow-up study with low-intensity use: twice a week for 30-min sessions for 3 more weeks, then returned to a follow-up assessment, in which they completed the psychological questionnaires again. During the post-intervention assessment, the control group received access to the RMPY-008 app for their free use. The study was approved by the IDC Institutional Review Board (IRB) (No. P_2023173). The neuroimaging study protocol was reviewed and approved by the IRB of Sheba Medical Center (No. 8591-21-SMC). All research was performed according to the relevant guidelines and regulations and complied with the Declaration of Helsinki.

### The digital treatment

The participants utilized a comprehensive digital treatment mobile application developed by Remepy Health Ltd. (Ramat Gan, Israel). The RMPY-008 application provides a structured and tailored daily treatment protocol. For 21 days, the participants received digitized and simplified psychological interventions with techniques drawn from Mindfulness-Based Stress-Reduction (MBSR), Mindfulness-Based Cognitive Therapy (MBCT), Attention Training Technique (ATT), Acceptance and Commitment Therapy (ACT), cognitive behavioral therapy (CBT) and psychoeducation, all of which have been shown to address psychological conditions such as stress, depression and anxiety^36–40^. These were delivered through short interactive videos, audio, and interactive games. The daily activities in the psychological interventions part of the program are listed in Supplementary Table 11. Participants that opted in for the additional follow up period used the app twice a week for an additional 3 weeks, the daily activities in the psychological interventions during the follow up are listed in Supplementary Table 12. Sample screens are shown in Supplementary Fig. 5. In addition to the psychological part of the intervention, the RMPY-008 app also features a virtual spatial navigation exercise as described previously^52,53^. Briefly, the task is based on sensorimotor integration and visual masking. Using digital 3D Hebb-Williams mazes, participants progress through three training phases: fully sighted navigation with auditory cues, a partially masked 3D phase, and a final blindfolded phase relying on auditory feedback. This approach was found to strengthen brain connectivity, spatial learning, and sensory-cognitive integration. The RMPY-008 application also included a daily notification to remind the participants to access the app and the daily activity. The psychological part of the daily interaction lasted about 20 min a day, and the navigation part of the daily interaction lasted about 10 min a day. If a participant missed a daily activity, there was an option to catch up and complete it later. All the completed daily activities could be accessed via a content library at any time (see Supplementary Fig. 5E).

### Psychological evaluation questionnaires

Changes in the subject’s psychological state were assessed by standardized psychological questionnaires: the Perceived Stress Scale (PSS-10)^110^, Brief State and Trait Anxiety Inventory (STAIT-5/STAIS-5)^108^, the Center for Epidemiologic Studies Depression Scale (CES-D)^111^, Mental Health Continuum Short Form (MHC-SF)^112^ and the Brief Resilient Coping Scale (BRCS)^113^.

### Measurement of cytokine levels using LEGENDplex assay

Blood samples were centrifuged for 10 min at 1600 g, room temperature. A serum supernatant was collected and centrifuged for 10 min at 14000 g, 4 °C divided into aliquots, and frozen at -80 °C until further analysis was performed.

Serum samples were diluted at a 1:1 ratio, and cytokines/cheomkines levels were determined using the LEGENDplex™ Human Inflammation Panel I (BioLegend, 740809) following the manufacturer’s protocol. Measurements were performed with a Cytek® Aurora flow cytometer, concentrations and the Limit of Detection (LOD) for each cytokine were calculated using BioLegend’s LEGENDplex^TM^ data analysis software.

### Brain imaging

A subset of 27 participants from the test group and 12 participants from the control group underwent additional structural and functional brain imaging. Brain imaging MRI scans were performed on MAGNETOM Prisma 3 T Scanner, configured with a 64-channel receiver head coils (Siemens Healthcare, Erlangen, Germany), at the Ruth and Meir Rosental Brain Imaging Center (MRI), Reichman University. The MRI protocol included the following sequences: Two runs of 300 volumes (9:28 min) resting state fMRI scans were acquired using a multi-band echo planar imaging sequence, CMRR EPI 2D^114,115^. Scan parameters: TR: 1870 ms, TE: 30 ms, flip angle: 75°, voxel size: 3.0 × 3.0×2.0 mm, FOV: 192, number of slices: 58 axial slices parallel to the AP-PC plane. During scanning, each participant was asked to remain still and relaxed, with their eyes fixated on a cross, and without thinking of anything deliberate. Foam pads and earplugs were employed to reduce head motion and scanning noise. Structural T1-weighted MRI scans were acquired for co-registration purposes using a T1-weighted 3D magnetization-prepared rapid gradient-echo (MPRAGE) sequence in a sagittal plane with 1 mm isotropic resolution. Sequence parameters: TR: 2000 ms, TE: 1.9 ms, flip angle: 9°, TI: 920 ms, FOV: 256 × 256, and 176 contiguous slices. The MRI protocol also included T2-Fluid-attenuated inversion recovery (FLAIR) sequence, using standard parameters for clinical brain evaluation.

Functional connectivity analysis was carried out using the CONN-fMRI toolbox v22a as implemented using statistical parametric mapping software SPM12 (http://www.fil.ion.ucl.ac.uk/spm). Functional volumes pre-processing pipeline included realignment with correction of susceptibility distortion interactions, slice timing correction, outlier detection, direct segmentation, and MNI-space normalization, with a resolution voxel size of 2.0 × 2.0 × 2.0 mm, and spatial smoothing (8 mm FWHM Gaussian kernel) steps^116^. The preprocessing steps derived (1) the realignment covariate, containing the six rigid-body parameters characterizing the estimated subject motion, (2) the scrubbing covariate containing potential outlier scans performed with CONNs artifact detection tool (ART), and (3) the quality assurance (QA) covariate based on global signal change (>3 standard deviations from the mean image intensity) and framewise displacement (FD) scan-to-scan head-motion. Age and sex were also used as group (second level) covariates. A component-based noise correction procedure (CompCor) approach^117^ was used to identify additional confounding temporal factors controlling for physiological noise, BOLD signal present in white matter, and head motion effects. Finally, residual BOLD time series were then bandpass-filtered at a frequency range of 0.009–0.1 Hz^116^. Individual seed-based connectivity maps (SBC) were were estimated characterizing the patterns of functional connectivity with pre-defined ROI’s (HCPex atlas), a modified and extended version of the Human Connectome Project-MultiModal Parcellation atlas (HCP-MMP)^118^. To test our hypotheses, we examined rsFC, using key limbic brain structures derived from the HCPex: the insula (thirteen subdivisions forming the insular frontal operculum cortical division)^119^, the amygdala and the hippocampus. Bivariate correlation analysis was used to determine the linear association of the BOLD time series between the seed and significant voxel clusters. Fisher’s Z transformation was applied to the correlation coefficients to satisfy normality assumptions. Then, functional connectivity maps were thresholded at *p* < 0.05 family wise error (FWE) corrected for multiple comparisons. Finally, participants with head motions of >2 mm in any direction between volumes, rotations of >2° in any axis during scanning, or mean FD of >0.5 in either the pre- or post-treatment maps were excluded from the dataset.

### Application adherence and user experience

Adherence to the RMPY-008 application was calculated using two measures: (1) the number of days of engagement with the application per week, and (2) the percentage of completion of the daily activities. The participants were asked to provide their feedback on the application using a questionnaire that covered their level of adherence and satisfaction with different aspects of the application. A description of the questionnaire is provided in Supplementary Table 10.

### Statistical analysis

#### Descriptive statistics

Baseline characteristics of the control and test groups were analyzed to compare demographic and clinical variables. For continuous variables (e.g. age, years of education, etc.), group means, and standard deviations were calculated, expressed as mean ± SD. Categorical variables, such as marital status, were reported as proportions for each category (e.g., married, divorced, widower, single), while binary variables, such as gender and self-reported anxiety or depression, were also summarized as proportions within each group. To assess differences between groups, we applied independent sample t-tests to continuous variables, comparing means between the control and test groups. For binary and categorical variables, chi-square tests of independence were used to test for significant differences in proportions.

#### Psychological questionnaires

Psychological questionnaires were analyzed using a linear mixed-effects model (LMM) with fixed effects for group (control vs. test) and time (pre vs. post intervention) to assess temporal changes, and random effects to capture inter-subject variability. Planned post-hoc comparisons were conducted using *t*-tests to elucidate specific time-dependent differences within each group, and between groups. Effect sizes were reported using Cohen’s d and using the Pre-Post-Control (PPC) design for the LMM interaction effect.

#### Cytokines

Cytokine levels were analyzed across time points, with data pre-processed to exclude outliers (defined as values > 3*Median Absolute deviation (MAD) and values below the limit of detection (LOD) to ensure high data quality (see Supplementary Table 6 for number of samples in each analysis)). A linear mixed-effects model (LMM) was applied to account for repeated measurements within subjects, including fixed effects for group (control vs. test) and time (pre vs. post intervention) to assess temporal changes, and random effects to capture inter-subject variability. Planned post-hoc comparisons were conducted using *t*-tests to elucidate specific time-dependent differences within each group, and between groups. Effect sizes were reported using Cohen’s d and using the Pre-Post-Control (PPC) design for the LMM interaction effect. To compare the cumulative distributions of normalized cytokine levels between test and control groups, Mann-Whitney (MW) U test was performed.

#### Correlation analysis

Pairwise correlations were analyzed separately for the test and control groups using the Pearson correlation coefficients, accompanied by p-values obtained through individual significance tests for each variable pair. Sample size (N) for each pairwise correlation was determined based on the number of valid (non-missing) observations per variable pair. To validate that the difference between groups is not due to the differences in sample size, we repeated the correlation analysis using randomly chosen 20 sub-populations of subjects from the test group, with equal size to the control group.

For the correlation diagram, a Multidimensional Scaling (MDS) approach was employed to project the correlation distances into a two-dimensional space, preserving relative similarities between variables. Network graphs were constructed using significant correlations as edges, such that edge thickness represents the strength of correlation, edge color varies from blue (negative correlation) to red (positive correlation) and node size reflects the overall correlation strength of each variable.

All p-values (Psychological questionnaires and Cytokines) were adjusted for multiple comparisons using the false discovery rate (FDR) correction with the Benjamini-Hochberg method to control for type I error. All statistical analyses were performed in R and Python, with modeling suited to the dataset’s structure and repeated-measures design.

#### Imaging analysis statistics

At the group level, SBC rsFC and t-fMRI individual maps were analyzed using the mixed design repeated measure ANOVA model to test the main interaction effect between time and group. RsFC is considered significant at joint-probability thresholds of 0.001 at the voxel level, and *p* < 0.05 family-wise error (FWE) corrected for multiple comparisons across the whole brain at the cluster level, with a minimum cluster size of 50 voxels. A bivariate group-level regression analysis with non-imaging covariates (e.g. psychological data, blood tests) covariate model was used to identify global brain correlations. The analysis will be implemented in SPM12 software (http://www.fil.ion.ucl.ac.uk/spm) with a parametric analysis approach across the entire brain volume^116^. RsFC correlation was considered significant at joint-probability thresholds of 0.001 at the voxel level, and *p* < 0.05 FDR procedure corrected for multiple comparisons across the whole brain at the cluster level, with a minimum cluster size of 50 voxels. The REX toolbox was used to extract cluster statistical values and individual rsFC data^116^. Then, the spearman rank correlation was used to test for associations with the non-imaging covariates.

## Supplementary information

Supplementary Information

## Data Availability

The data that support the findings of this study are available from the corresponding author upon reasonable request.
