# Supplementary material for: Mobile application leads to psychological improvement and correlated neuroimmune function change in subjective cognitive decline
Source: NPJ Digit Med. 2025 Jun 14;8:359. doi: 10.1038/s41746-025-01765-1 (PMC12167371; doi:10.1038/s41746-025-01765-1)
Supplement: Supplementary file 1 — Supplementary Information [file 41746_2025_1765_MOESM1_ESM.pdf]

Mobile Application Leads to Psychological Improvement and Correlated Neuroimmune Function Change in Subjective Cognitive Decline

Merav Catalogna[1, †], Nira Saporta[2, †], Bar Nathansohn-Levi[2], Tal Tamir[2], Ariel Shahaf[2], Shira Molcho[2], Shai Erlich[2], Shahar Shelly[3,4], and Amir Amedi[1 *]

Supplementary Information

1 SUPPLEMENTARY INFORMATION 2
1.1 Eligibility Criteria 2
1.2 The spatial navigation training program 3
2 SUPPLEMENTARY TABLES 4
Table 1: The psychological intervention in the program: Follow-up low intensity period 4
Table 2: Questionnaire reliability analysis 5
Table 3: Questionnaire Analysis 5
Table 4: Correlations between change in questionnaires 6
Table 5: Questionnaire Analysis – Follow-up period 6
Table 6: Immune Mediators Analysis 7
Table 7: Correlations between change in cytokines levels 7
Table 8: Longitudinal changes in connectivity measures: Test>Ctrl; Post>Pre-intervention 8
Table 9: Significant association between alterations in brain connectivity and change in cytokine levels 8
Table 10: Application adherence and satisfaction questionnaire 10
Table 11: The psychological intervention in the program 11
Table 12: The psychological intervention in the program: Follow-up low intensity period 12
3 SUPPLEMENTARY FIGURES 13
Figure 1: Study flowchart 13
Figure 2: Proportion of participants by change in cytokine level 14
Figure 3: Correlation network diagram of immune mediators in the control group 15
Figure 4: Post-intervention alterations in the test group. 16
Figure 5: Remepy-008 APP mobile application 17
4 REFERENCES 18

1 Supplementary Information

1.1 Eligibility Criteria

Inclusion criteria

1. Age 50-65 years at the time of signing the informed consent form.
2. Hebrew speakers:
3. Self-reporting to be healthy and free from chronic physical or mental illnesses.
4. Subjectively report of memory decline and a state of increase stress or anxiety
5. At Screening, had a STAIT-5 score of ≥ 10.
6. Normal (or corrected to normal) Hearing and Vision
7. Able to understand and sign the informed consent form.

Exclusion criteria

1. Known to have a history of brain injury or neurodegenerative disorder.
2. Know another, concurrent and active, neurological disease, or is known to have a significant neurological disease such as, but are not limited to: Parkinson's disease, Multiple sclerosis, Epilepsy, Huntington's disease, Amyotrophic lateral sclerosis (ALS), Cerebral palsy, and Stroke, etc.
3. Known to have a serious psychiatric disorder such as post-traumatic stress disorder, bipolar disorder, schizophrenia and/or psychosis.
4. Known regular user (including "recreational use") of any illicit drugs at the time of signing informed consent or has a history within the last year of substance abuse including (but are not limited to): alcohol, Cocaine, Crack cocaine, Ecstasy, Hallucinogens (such as LSD, PCP, mushrooms, and Salvia), Heroin, Inhalants (such as spray paints, markers, cleaning supplies), Ketamine, Marijuana, Meth, Synthetic marijuana, etc.
5. Major medical comorbidity, such as (but not limited to) severe heart/renal failure (ejection fraction <20%, Cr>2.5), hepatic failure, severe respiratory disease, uncontrolled diabetes mellitus (HbA1c% > 8.5%).
6. Active infectious disease in the 30 days prior to enrollment
7. Active malignancy or malignancy treatment with chemotherapy, radiation, or immuno-oncology drugs in the two years prior to enrollment
8. Known not to have a balanced thyroid-stimulating hormone level while on thyroid hormone replacement therapy (TSH levels > 10 U).
9. Is unable or unwilling to complete the study assessments.
10. MRI contraindication or claustrophobia (MRI subset)
11. Recent (past 30 days) loss of first degree relative
12. Use of the following medications or treatments:

a. Chronic use of antidepressants including SSRIs, SNRIs, NDRIs, TCAs.
b. Chronic or intermittent use of benzodiazepines
c. Has regularly used of opioids, which include, but are not limited to morphine, codeine, oxycodone, hydrocodone, fentanyl, tramadol, and methadone, more than three times per week in the 30 days prior to enrollment.
d. Chronic use of systemic steroids > 5 mg prednisone per day or its equivalence. Subjects with asthma who require intermittent use of bronchodilators, inhaled steroids, or local steroid injections will not be excluded from the study.
e. Chronic use of non-specific adrenergic blockade treatment such as propranolol, labetalol, sotalol, carvedilol, timolol, nadolol, pindolol, penbutolol.
f. Previous treatment received within the last 6 months with any of the following:
g. Approved or investigational immune-oncology agent; or,
h. Immunosuppressants; or,
i. Immunostimulants; or,
j. Vaccines, including influenza and covid vaccines, in the 30 days prior to enrollment and during the study period (approximately 4 weeks)

k. Participation in, or completion of, a program or study related to mindfulness, CBT, or stress reduction in the last 3 months.
l. Engagement in mindfulness exercises and/or meditation more than 5 minutes daily or 60 minutes weekly (either using a smartphone or not) for the last 3 months.

1.2 The spatial navigation training program

The virtual spatial navigation training was based on a set of Hebb-Williams mazes. This virtual spatial navigation exercise uses principles of sensory integration (using more than one sense), sensory deprivation (blocking input to an otherwise normally used sense) and sensory substitution (providing information normally provided to one sense via another sense) [1]. The virtual navigation training protocol employs an innovative, integrative approach to spatial memory and navigation training, designed to accelerate the learning process and induce sensory and cognitive network balance [2,3]. The unique approach embedded in the RMP-008 app combines both egocentric (a self-centered navigation, that involves encoding spatial information from the perspective of the navigator) and allocentric (relies on the navigator's perception of landmark positions relative to one another, forming a cognitive map) navigation strategies through a three-step blindfold training protocol, with progressively increasing navigation complexity: each new maze trial starts with a top view map of the maze followed by a sighted virtual 3D navigation experience, which also includes auditory input regarding the distance from the walls. In the next step, navigation becomes more challenging as 50% of the maze is randomly clouded, reducing half of the visual cues and encouraging subjects to integrate visual cues with the auditory cues as well as relying more on their memory, and in the final step, participants are asked to navigate the maze blindfolded, relying on spatial memory and auditory cues alone (Fig. 2 F-I). While the main task was successful wayfinding, participants were instructed to find the fastest route to the exit while avoiding collisions with the walls. The distance audio algorithm utilizes a sound frequency conversion where a higher frequency indicates proximity to a nearby wall, while a lower frequency signifies greater distance from the wall. Footstep sounds signal a clear passage. Participants guided their way by swiping their fingers across the touchscreen in eight 45-degree directions. The software automatically logged errors, time, and performance for each session.

2 Supplementary Tables

Supplementary Table 1: The psychological intervention in the program: Follow-up low intensity period

Day	Name of Interventions	Explanation
Module: Dealing with discomfort and difficulty		
1	Dealing with discomfort and difficulty intro	A video introducing the principles of dealing with discomfort and difficulty, followed by a video introducing the "second arrow of suffering parable", which focuses on dealing with suffering more skillfully, followed by another practice of mindful breathing
2	The flexible brain	A video explaining the principles of neuroplasticity, followed by A video introducing the concept of the struggle switch, which focuses on the suffering caused by struggling with emotional or physical discomfort and what can be done to reduce it, followed by positive experience monitoring activity
3	3-step breathing space	A video explaining the principles of a mindfulness practice called the 3-step breathing space, followed by audio guidance for 3-step breathing space, and a video explaining how to use this practice in daily life
4	The white bear	A video introducing the concept of the white bear, which focuses on the difficulty of intentionally pushing thoughts away from our mind, followed by identification of thought distortions activity, followed by another practice of safe place guided imagery
5	Avoidance, struggle and acceptance	A video introducing the concept of acceptance as an alternative to avoidance or struggle, followed by another practice of grounding
6	The RAIN model	A video introducing the concept of the RAIN model (Recognize, Allow, Investigate and Natural awareness), which is a mindfulness practice, followed by another practice of relaxation guided imagery and a summary of the last module

Supplementary Table 2: Questionnaire reliability analysis

Questionnaire	N (Number of items)	Cronbach's α
**PSS**	10	.86
**CES-D**	20	.87
**MHC-SF**	14	.92
**BRCS**	4	.71
**STAIT-5**	5	.74
**STAIS-5**	5	.87

BRCS- Brief Resilient Coping Scale, CES-D- Center for Epidemiological Studies Depression, MHC-SF- Mental Health Continuum Short Form, PSS- Perceived Stress Scale, STAIT-5- Trait Anxiety, STAIS-5- State Anxiety

Supplementary Table 3: Questionnaire Analysis

	Pretest		Posttest		LMM interaction effect		Planned Post hoc t-test				
	**M**	**SD**	**M**	**SD**	**p**	**Effect Size (PPC)**	**t**	**df**	**p**	**Cohen's d**	**% change**
**PSS Control**	22.44	4.93	20.22	5.88	0.127	-0.35	2.72	31	0.066	-0.41	-9.89
**PSS Trial**	21.55	6.26	17.56	6.32			6.36	70	0.001	-0.63	-18.5
**CES-D Control**	26.44	9.52	24.09	9.6	0.036	-0.49	1.95	31	0.18	-0.25	-8.87
**CES-D Trial**	25.42	9.82	19.54	10.43			6.64	70	<0.001	-0.58	-23.16
**MHC-SF Control**	50.53	17.19	49.72	16.31	0.021	0.59	0.48	31	0.633	-0.05	-1.61
**MHC-SF Trial**	50.23	12.48	54.49	12.5			-4.39	70	<0.001	0.34	8.5
**BRCS Control**	15	2.44	14.53	2.84	0.006	0.77	1.45	31	0.266	-0.18	-3.12
**BRCS Trial**	14.63	2.64	15.63	2.61			-4.31	70	<0.001	0.38	6.83
**STAIT-5 Control**	11.28	2.58	10.78	3.32	0.325	-0.21	1.24	31	0.266	-0.17	-4.43
**STAIT-5 Trial**	11.35	3.25	10.3	3.08			3.19	70	0.002	-0.33	-9.31
**STAIS-5 Control**	13.16	3.54	12.62	4.14	0.034	-0.52	1.31	31	0.266	-0.14	-4.04
**STAIS-5 Trial**	12.87	3.85	10.85	4.1			5.47	70	<0.001	-0.51	-15.75

BRCS- Brief Resilient Coping Scale, CES-D- Center for Epidemiological Studies Depression, MHC-SF- Mental Health Continuum Short Form, PSS- Perceived Stress Scale, STAIT-5- Trait Anxiety, STAIS-5- State Anxiety All p values FDR corrected for multiple comparisons.

Supplementary Table 4: Correlations between change in questionnaires

Questionnaire 1	Questionnaire 2	Control Correlation	Control P Value	Trial Correlation	Trial P Value
	N	31		70	
**PSS**	**CES-D**	0.44	0.05	0.54	<0.001
**PSS**	**STAIS-5**	0.31	0.17	0.27	0.03
**PSS**	**STAIT-5**	0.21	0.36	0.34	<0.001
**PSS**	**MHC-SF**	-0.23	0.32	-0.42	0.0006
**PSS**	**BRCS**	-0.13	0.61	-0.18	0.13
**CES-D**	**STAIS-5**	0.35	0.15	0.45	<0.001
**CES-D**	**STAIT-5**	0.11	0.63	0.41	<0.001
**CES-D**	**MHC-SF**	-0.17	0.48	-0.25	0.04
**CES-D**	**BRCS**	-0.04	0.82	-0.08	0.53
**STAIS-5**	**STAIT-5**	0.27	0.25	0.30	0.01
**STAIS-5**	**MHC-SF**	-0.07	0.75	-0.50	<0.001
**STAIS-5**	**BRCS**	0.04	0.82	-0.14	0.24
**STAIT-5**	**MHC-SF**	-0.25	0.30	-0.36	<0.001
**STAIT-5**	**BRCS**	-0.33	0.17	-0.03	0.77
**MHC-SF**	**BRCS**	0.41	0.07	0.32	<0.001

BRCS- Brief Resilient Coping Scale, CES-D- Center for Epidemiological Studies Depression, MHC-SF- Mental Health Continuum Short Form, PSS- Perceived Stress Scale, STAIT-5- Trait Anxiety, STAIS-5- State Anxiety

Supplementary Table 5: Questionnaire Analysis – Follow-up period

Questionnaire	M Pre	SD Pre	M Post	SD Post	M Fo-U	SD Fo-U	d[1]	d[2]	d[3]	P value[1]	P value[2]	P value[3]
**PSS**	20.73	5.52	16.05	5.51	15.25	5.05	0.85	1.04	0.15	<0.001	<0.001	0.34
**CES-D**	24.05	9.03	17.18	8.85	14.91	9.4	0.77	0.99	0.25	<0.001	<0.001	0.026
**MHC-SF**	51.48	10.57	56.16	10.56	59.14	11.3	-0.44	-0.7	-0.27	0.002	<0.001	0.014
**BRCS**	14.91	2.51	15.95	2.38	16.45	2.43	-0.43	-0.62	-0.21	0.003	<0.001	0.066
**STAIT-5**	11.27	3.21	9.86	2.69	9.43	2.98	0.48	0.59	0.15	0.005	<0.001	0.28
**STAIS-5**	12.2	3.53	9.89	3.49	9.36	3.64	0.66	0.79	0.15	<0.001	<0.001	0.33

BRCS- Brief Resilient Coping Scale, CES-D- Center for Epidemiological Studies Depression, MHC-SF- Mental Health Continuum Short Form, PSS- Perceived Stress Scale, STAIT-5- Trait Anxiety, STAIS-5- State Anxiety, Fo-U – Follow up; d, Cohen's d; [1]Pre – Post, [2]Pre-Fo-U, [3]Post – Fo-U

Supplementary Table 6: Immune Mediators Analysis

	Pretest			Posttest		LMM interaction effect		Planned Post hoc t-test				
	N	M	SD	M	SD	p	Effect Size (PPC)	t	df	p	Cohen's d	% change
**TNF-a Control**	25	6.59	3.87	6.81	3.99	0.039	-0.53	-0.43	65	0.82	0.06	3.38
**TNF-a Trial**	42	8.57	4.89	7.41	4.54			2.9	65	<0.001	-0.25	-13.56
**IL-17 Control**	19	0.75	0.61	0.78	0.59	0.023	-0.73	-0.38	57	0.82	0.04	3.2
**IL-17 Trial**	40	0.88	0.62	0.7	0.5			4.1	57	<0.001	-0.32	-20.2
**IL-23 Control**	19	6.77	5.27	7.83	4.46	0.011	-0.82	-1.19	65	0.79	0.22	15.68
**IL-23 Trial**	48	10.35	5.69	8.25	4.53			3.76	65	<0.001	-0.41	-20.34
**MCP-1 Control**	29	640.82	264.24	684.96	260.69	0.008	-0.8	-1.47	72	0.79	0.17	6.89
**MCP-1 Trial**	45	745.6	333.04	660.76	285.77			3.51	72	<0.001	-0.27	-11.38
**IFN- γ Control**	20	5.66	4.12	5.81	4.22	0.035	-0.62	-0.24	58	0.82	0.04	2.68
**IFN- γ Trial**	40	7.71	4.64	6.06	4.14			3.61	58	<0.001	-0.37	-21.34
**IL-12 Control**	15	2.93	1.53	3.0	1.47	0.035	-0.7	-0.22	45	0.82	0.05	2.37
**IL-12 Trial**	32	4.09	2.29	3.32	1.76			3.65	45	<0.001	-0.38	-18.9

Supplementary Table 7: Correlations between change in cytokines levels

Variable 1	Variable 2	N Control	Control Correlation	Control P Value	N Trial	Trial Correlation	Trial P Value
**TNF -a**	**IL 17**	16	0.25	0.502	28	0.62	<0.001
**TNF -a**	**IL 23**	18	0.66	0.008	33	0.74	<0.001
**TNF -a**	**IFN- γ**	19	0.02	0.916	28	0.65	<0.001
**TNF -a**	**IL 12**	13	0.21	0.574	23	0.40	0.061
**TNF -a**	**MCP-1**	23	0.10	0.703	27	0.36	0.064
**IL 17**	**IL 23**	13	0.30	0.502	28	0.67	<0.001
**IL 17**	**IL 23**	13	0.30	0.191	28	0.67	<0.001
**IL 17**	**IL 12**	11	0.51	0.244	23	0.72	<0.001
**IL 17**	**MCP 1**	19	0.67	0.007	29	0.64	<0.001
**IL 23**	**IFN- γ**	15	0.22	0.550	27	0.73	<0.001
**IL 23**	**IL 12**	8	-0.09	0.878	21	0.56	0.009
**IL 23**	**MCP-1**	17	0.27	0.500	31	0.48	0.006
**IFN- γ**	**IL 12**	11	0.81	0.007	22	0.75	<0.001
**IFN- γ**	**MCP-1**	18	0.20	0.550	23	0.60	0.003
**IL 12**	**MC-1**	15	0.33	0.457	21	0.41	0.063

Supplementary Table 8: Longitudinal changes in connectivity measures: Test>Ctrl; Post>Pre-intervention

Seed ID*	Seed area	Peak cluster (x,y,z)	K	T	p-FWE	p-FDR	p-UNC	Brain area	BA
HCPex_2mm.250	INS r	44 32 38	474	5.44	0.0000	0.0000	<0.001	dlPFC r	9
HCPex_2mm.250	INS r	12 36 22	403	6.14	0.0000	0.0000	<0.001	dACC r	32
HCPex_2mm.250	INS r	-36 30 42	336	5.92	0.0000	0.0000	<0.001	dlPFC l	9
HCPex_2mm.250	INS r	68 -40 24	202	5.53	0.0004	0.0002	<0.001	SMG r	40
HCPex_2mm.250	INS r	16 56 8	165	5.05	0.0016	0.0006	<0.001	mPFC r	10
HCPex_2mm.78	INS l	-24 -34 -6	286	4.63	0.0001	0.0001	<0.001	Hipp l	
HCPex_2mm.78	INS l	30 -22 56	266	5.48	0.0001	0.0001	<0.001	PreSG r	6
HCPex_2mm.78	INS l	10 -16 52	183	4.70	0.0016	0.0010	<0.001	PreSG r	6
HCPex_2mm.78	INS l	38 -66 -2	144	4.15	0.0071	0.0032	<0.001	LOC r	19
HCPex_2mm.78	INS l	18 34 44	88	4.80	0.0730	0.0275	0.002	m PFC r	8
HCPex_2mm.420	Amyg r	-42 24 24	219	-5.78	0.0004	0.0003	<0.001	MidFG l,	44

* Glasser atlas [4], l, left, r, right, UNC, uncorrected, FDR, false discovery rate, FWE, family wise error, K, cluster size, BA, Brodmann area

Supplementary Table 9: Significant association between alterations in brain connectivity and change in cytokine levels

Cytokine	Seed to Area	Peak cluster (x,y,z)	r*	p-value	n
**IL-17**	AMYr-mPFC	24 64 -4	-0.55	0.007	22
**IL-17**	AMYr-SMG	-68 -24 30	0.49	0.018	22
**IL-17**	HIPPr-OFC	-16 12 -30	-0.45	0.032	22
**IL-17**	INSl-OFC	26 44 -6	-0.76	<0.001	22
**IL-17**	INSl-PORB	21 42 -12	-0.57	0.006	22
**IL-17**	INSr-HIPP	-24 -34 -6	0.47	0.027	22
**IL-17**	INSr-HIPP	-32 -14 -20	-0.52	0.012	22
**IL-17**	INSr-mPFC	-2 42 0	-0.60	0.003	22
**IL-17**	INSr-PSC	-40 -18 14	-0.63	0.002	22
**IL-23**	AMYl-aPFC	20 52 -10	-0.54	0.011	21
**IL-23**	AMYl-CAUD	10 22 2	-0.52	0.016	21
**IL-23**	AMYr-dlPFC	-34 26 20	-0.57	0.007	21
**IL-23**	AMYr-mPFC	24 64 -4	-0.55	0.010	21
**IL-23**	AMYr-OFC	-16 52 -22	-0.582	0.006	21
**IL-23**	AMYr-PREC	24 -66 38	0.49	0.025	21
**IL-23**	INSl-OFC	26 44 -6	-0.58	0.005	21
**IL-23**	INSl-PORB	-44 32 -14	-0.68	0.001	21
**IL-23**	INSr-ACC	-4 38 4	-0.69	<0.001	21
**IL-23**	INSr-HIPP	34 -18 -20	-0.47	0.029	21
**IL-23**	INSr-HIPP	-32 -14 -20	-0.72	<0.001	21
**IL-23**	INSr-mPFC	-2 42 0	-0.69	0.001	21
**IL-23**	INSr-PSC	-40 -18 14	-0.73	<0.001	21
**IL-23**	INSr-THAL	18 -28 18	0.59	0.005	21
**TNF-a**	INSl-OFC	26 44 -6	-0.43	0.037	23
**TNF-a**	INSl-PORB	-44 32 -14	-0.42	0.043	23

**TNF-a**	INSr-ACC	-4 38 4	-0.60	0.002	23
**TNF-a**	INSr-HIPP	34 -18 -20	-0.46	0.026	23
**TNF-a**	INSr-mPFC	-2 42 0	-0.66	0.001	23
**TNF-a**	INSr-mPFC	4 36 8	-0.54	0.007	23
**TNF-a**	HIPPr-BROCA45	-42 26 6	-0.55	0.007	23
**IFN-g**	AMYl-mPFC	0 52 38	-0.61	0.003	22
**IFN-g**	AMYr-mPFC	24 64 -4	-0.67	0.001	22
**IFN-g**	AMYr-PREC	18 -64 38	0.53	0.011	22
**IFN-g**	AMYr-PREC	24 -66 38	0.47	0.025	22
**IFN-g**	AMYr-SMG	-62 -38 26	0.48	0.024	22
**IFN-g**	INSl-OFC	26 44 -6	-0.55	0.008	22
**IFN-g**	INSl-PORB	21 42 -12	-0.49	0.020	22
**IFN-g**	INSr-HIPP	-32 -14 -20	-0.53	0.010	22
**IFN-g**	INSr-PSC	-40 -18 14	-0.47	0.025	22
**IFN-g**	INSr-THAL	18 -28 18	0.71	<0.001	22
**IL-12**	AMYl-CAUD	10 22 2	-0.77	<0.001	19
**IL-12**	AMYr-mPFC	24 64 -4	-0.64	0.003	19
**IL-12**	AMYr-OFC	-16 52 -22	-0.55	0.014	19
**IL-12**	AMYr-SMG	-62 -38 26	0.71	0.001	19
**IL-12**	AMYr-SMG	-68 -24 30	0.51	0.024	19
**IL-12**	INSl-OFC	26 44 -6	-0.46	0.045	19
**IL-12**	INSr-HIPP	-32 -14 -20	-0.48	0.034	19
**MCP1**	AMYr-CAUD	-20 12 16	0.66	0.005	16
**MCP1**	AMYr-mPFC	24 64 -4	-0.52	0.035	16
**MCP1**	AMYr-SMG	-68 -24 30	0.72	0.001	16
**MCP1**	HIPPr-OFC	-16 12 -30	-0.68	0.003	16
**MCP1**	INSr-HIPP	-24 -34 -6	0.62	0.010	16
**MCP1**	INSr-PSC	-40 -18 14	-0.62	0.010	16

* r, Pearson's correlation coefficient; n, number of patients; Amygdala, AMY, Insula, INS, Hippocampus, HIPP, medial prefrontal cortex, mPFC, Suppermarginal gyrus, SMG, Orbitofrontal cortex, OFC, Paras Orbitalis, PORB, Primary sensory cortex, PSC, Precuneus, PREC, Anterior cingulate cortex, ACC, Thalamus, THAL, Caudate, CAUD, l, left, r, right

Supplementary Table 10: Application adherence and satisfaction questionnaire

Question	1	2	3	4	5
I completed all the activities	2.8%	0.0%	1.4%	26.8%	69.0%
The rationale and importance of the activities were clear	2.8%	0.0%	8.5%	35.2%	53.5%
The activities were well-organized and clear	0.0%	0.0%	0.0%	21.1%	78.9%
The activities were difficult	35.2%	26.8%	21.1%	14.1%	2.8%
The content was valuable to me	0.0%	2.8%	5.6%	43.7%	47.9%
I enjoyed the different activities	0.0%	4.2%	12.7%	36.6%	46.5%
During the different activities I learned new things	1.4%	4.2%	8.5%	43.7%	42.3%
I felt connected with the various contents in the program	0.0%	2.8%	2.8%	46.5%	47.9%
I felt that the content suited my needs	0.0%	4.2%	12.7%	42.3%	40.8%
I felt that using the app contributed to my health	0.0%	12.7%	32.4%	32.4%	22.5%
I would like to continue using this app when the experiment is over	0.0%	7.0%	15.5%	28.2%	49.3%
I would recommend this app to a friend	1.4%	5.6%	9.9%	32.4%	50.7%

Supplementary Table 11: The psychological intervention in the program

Day	Name of Interventions	Explanation
Module: Welcome onboard		
0	Welcome onboard	A brief introduction to the program (shown during baseline visit 2 on site after the app was installed on the participant's phone)
Module: Dealing with stress and anxiety		
1	Introduction and Progressive muscle relaxation	A brief introduction to the module, followed by a video introducing the principles and benefits of progressive muscle relaxation, then an audio guidance for progressive muscle relaxation
2	Safe place guided imagery	A video introducing the principles and benefits of guided imagery, followed by an audio of safe place guided imagery
3	Anxiety psychoeducation – part 1	A video introducing psychoeducation about anxiety and its physical symptoms, followed by a symptom identification activity and then another practice of the progressive muscle relaxation audio
4	Anxiety psychoeducation – part 2	A video introducing more psychoeducation about anxiety and its dynamics, followed another practice of the safe place guided imagery audio
5	Diaphragmatic breathing	A video introducing the principles and benefits of diaphragmatic breathing, followed by another practice of the progressive muscle relaxation audio
6	Living in the present and grounding	A video introducing the principles and benefits of grounding exercises, followed by an audio of a grounding exercise and then another practice of diaphragmatic breathing
7	Summary – dealing with stress and anxiety	A short summary video followed by a reflection on the first module and another practice of the safe place guided imagery audio
Module: Strengthening Myself		
8	Monitoring positive experiences	A video introducing the principles and benefits of monitoring positive experiences, followed by positive experience monitoring activity and another practice of the grounding audio
9	Self-validation	A video introducing the principles and benefits of Self-validation, followed by a self-validation activity and another practice of the progressive muscle relaxation audio
10	Self-compassion	A video introducing the principles and benefits of Self-compassion, followed by a self-compassion activity and another practice of the safe place guided imagery audio
11	Relaxation guided imagery	A video introducing the principles and benefits of relaxation guided imagery, followed by an audio of relaxation guided imagery
12	Summary – strengthening myself	A short summary video followed by a reflection on the second module and another practice of the relaxation guided imagery audio
Module: Attention and Autopilot		
13	Attention and Autopilot – intro	A video introducing the principles of attention control and autopilot mode, followed by another practice of the progressive muscle relaxation audio
14	Attention training	A video introducing the principles and benefits of attention training followed by an audio of attention training – the participant is requested to focus their attention on one auditory stimulus (e.g., sound of a waterfall) and then shift their focus to another auditory stimulus (e.g., sound of a bird).
15	About Mindfulness	A video introducing the principles and benefits of mindfulness, followed by attention training practice
16	Mindful breathing	A video introducing the principles and benefits of mindful breathing, followed by an audio guidance for mindful breathing and another practice of the progressive muscle relaxation audio

17	Mindful eating and summary	A video introducing the principles and benefits of mindful eating, followed by an audio guidance for mindful eating, followed by a short summary video of the third module and another practice of mindful breathing
Module: living inside your head		
18	Thoughts and interpretations	A video explaining the relation between interpretations of experiences and the emotional reactions to them, followed by an audio guidance for mindful breathing and another practice of attention training
19	Unhelpful thinking styles	A video explaining what unhelpful thinking styles and thought distortion patterns are, followed by identification of thought distortions activity, followed by another practice of relaxation guided imagery
20	Balancing thoughts	A video explaining how to balance distorted thoughts, followed by balancing thoughts activity, followed by another practice of progressive muscle relaxation
21	Thought distortions and anxiety + summary	A video explaining the connection between thought distortions and anxiety
End of study	Summary	A summary and farewell video

Supplementary Table 12: The psychological intervention in the program: Follow-up low intensity period

Day	Name of Interventions	Explanation
Module: Dealing with discomfort and difficulty		
1	Dealing with discomfort and difficulty intro	A video introducing the principles of dealing with discomfort and difficulty, followed by a video introducing the “second arrow of suffering parable”, which focuses on dealing with suffering more skillfully, followed by another practice of mindful breathing
2	The flexible brain	A video explaining the principles of neuroplasticity, followed by A video introducing the concept of the struggle switch, which focuses on the suffering caused by struggling with emotional or physical discomfort and what can be done to reduce it, followed by positive experience monitoring activity
3	3-step breathing space	A video explaining the principles of a mindfulness practice called the 3-step breathing space, followed by audio guidance for 3-step breathing space, and a video explaining how to use this practice in daily life
4	The white bear	A video introducing the concept of the white bear, which focuses on the difficulty of intentionally pushing thoughts away from our mind, followed by identification of thought distortions activity, followed by another practice of safe place guided imagery
5	Avoidance, struggle and acceptance	A video introducing the concept of acceptance as an alternative to avoidance or struggle, followed by another practice of grounding
6	The RAIN model	A video introducing the concept of the RAIN model (Recognize, Allow, Investigate and Natural awareness), which is a mindfulness practice, followed by another practice of relaxation guided imagery and a summary of the last module

3 Supplementary Figures

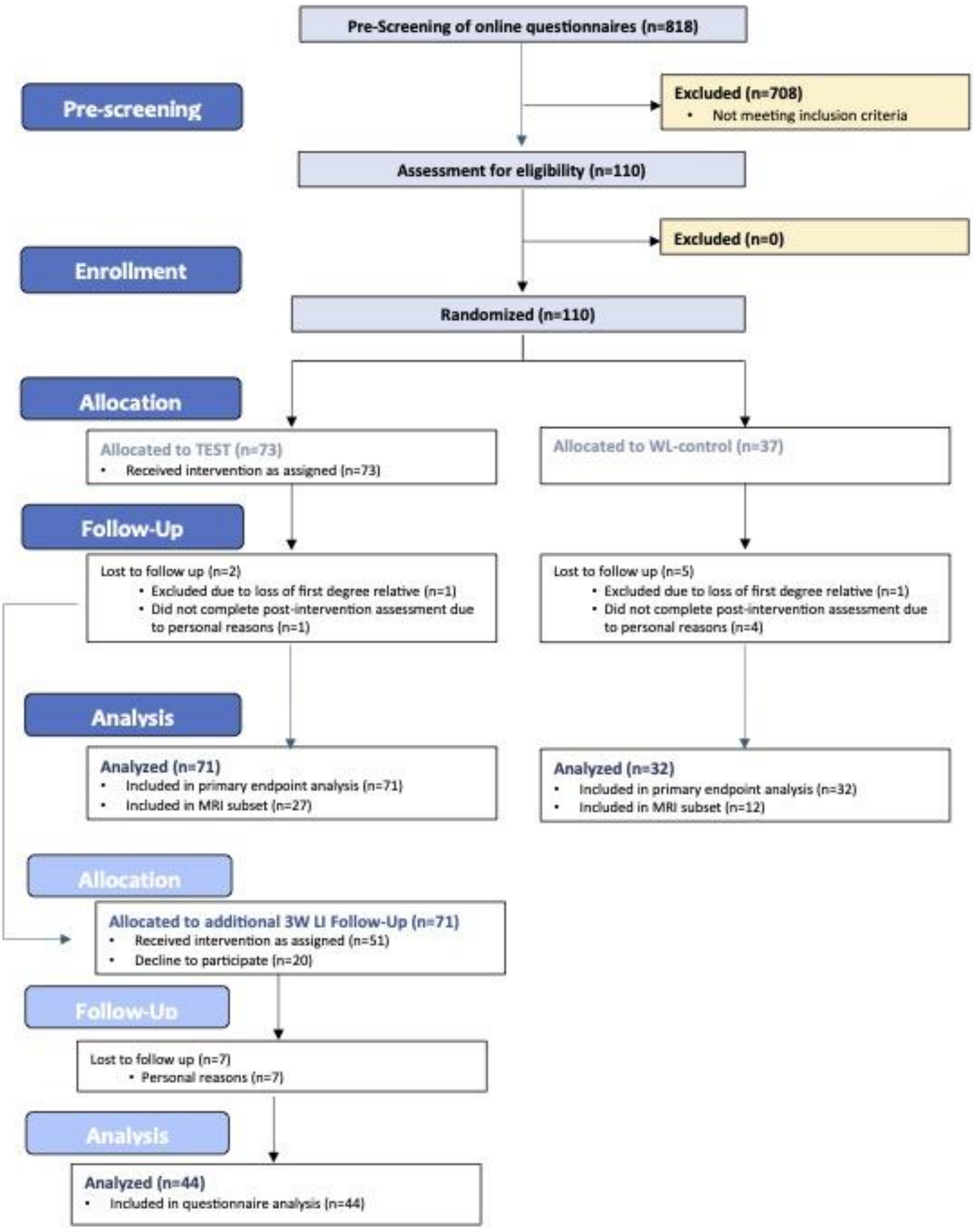

Supplementary Figure 1: Study flowchart

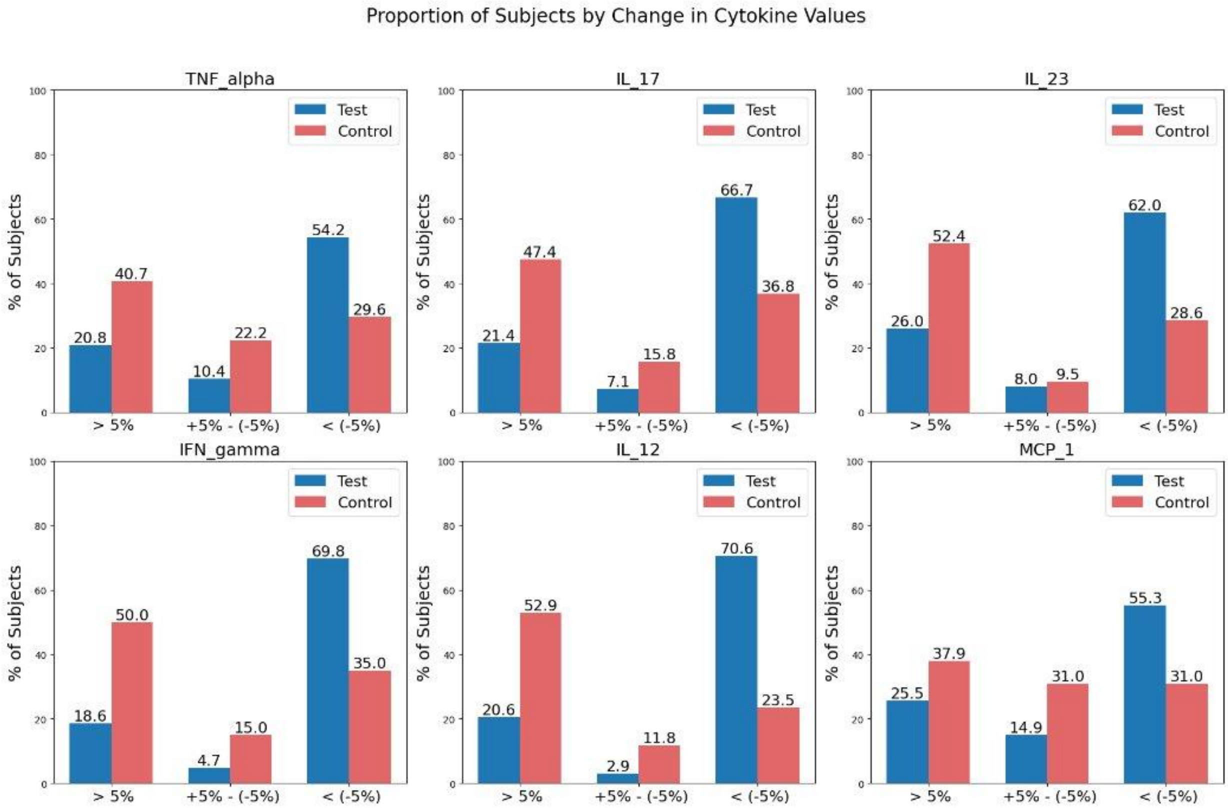

Supplementary Figure 2: Proportion of participants by change in cytokine level. For each cytokine the left bars indicate participants for which there was an increase greater than 5%, divided by group, the middle bars indicate participants for which there was an increase or a decrease smaller than 5%, divided by group, and the right bars indicate the participants for which there was a decrease greater than 5%, divided by group.

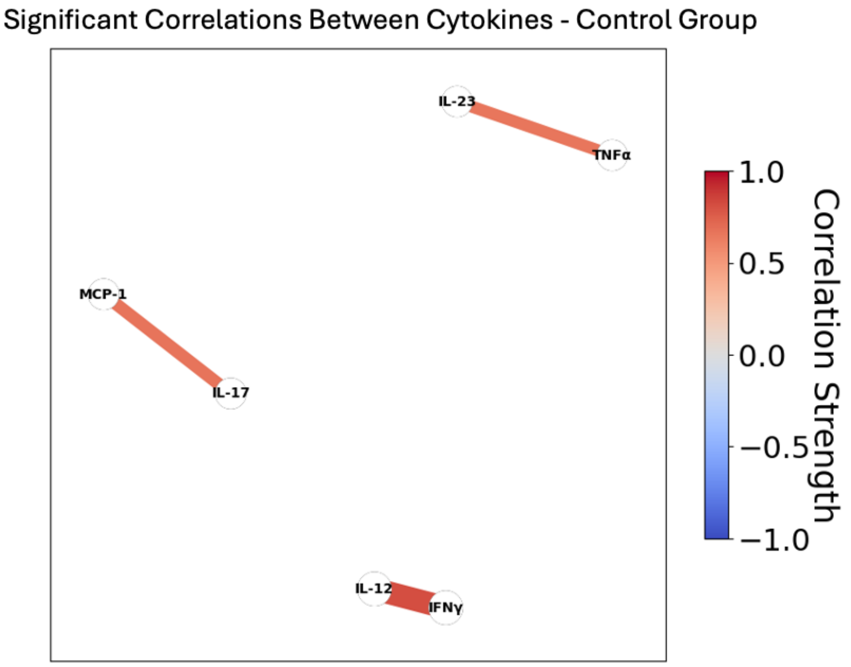

Supplementary Figure 3: Correlation network diagram of immune mediators in the control group. Nodes represent individual cytokines, with edges indicating significant correlations between individual changes from pre to post intervention ($p < 0.05$, FDR corrected for multiple comparisons). Edge thickness corresponds to the strength of the correlation, while edge color represents the correlation direction, ranging from blue (negative correlation, -1.0) to red (positive correlation, +1.0). Larger nodes indicate cytokines with higher overall correlation strength (See methods)

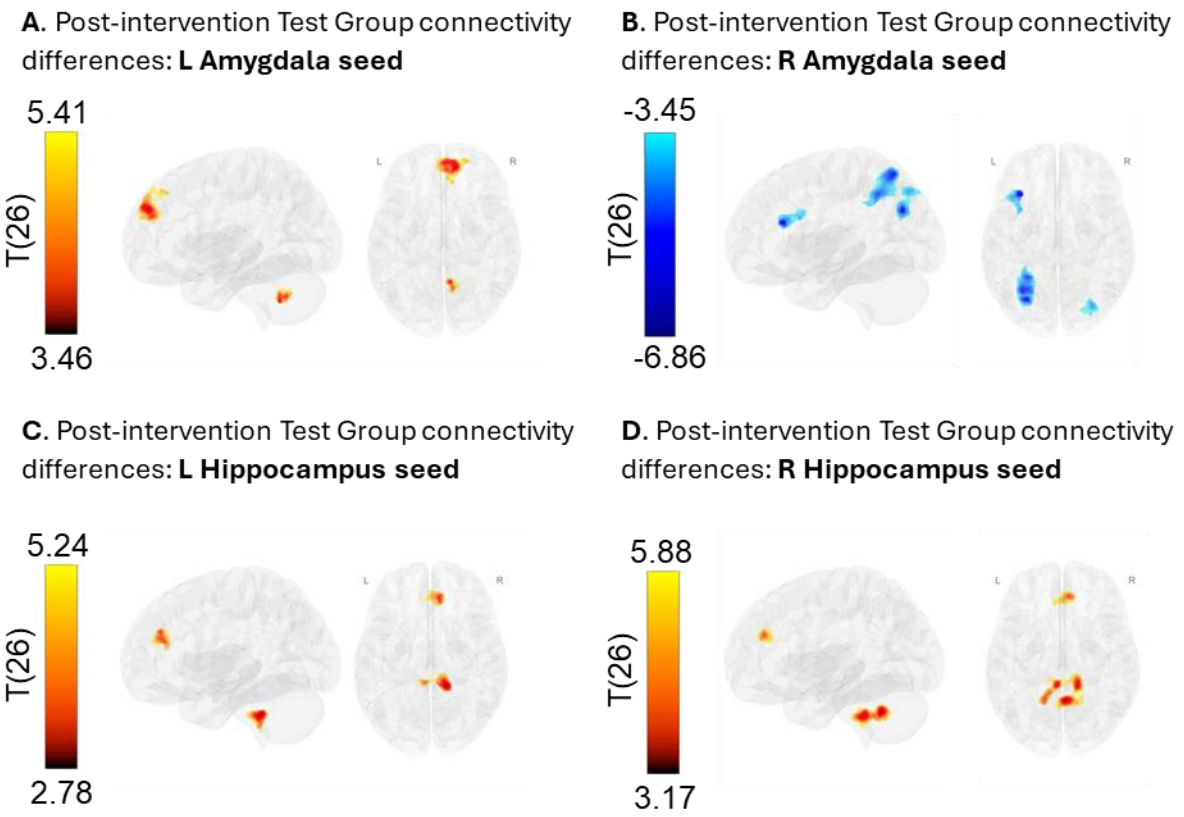

Supplementary Figure 4: Post-intervention alterations in the test group. Seed-to-voxel maps of longitudinal test group differences in A-B. the right and left amygdala seeds C-D. the left and right hippocampus seeds (voxel level, $p < 0.001$, cluster level, $p < 0.05$, FDR corrected, n = 27). Brain images were generated using CONN (RRID:SCR_009550) https://web.conn-toolbox.org, v22a.

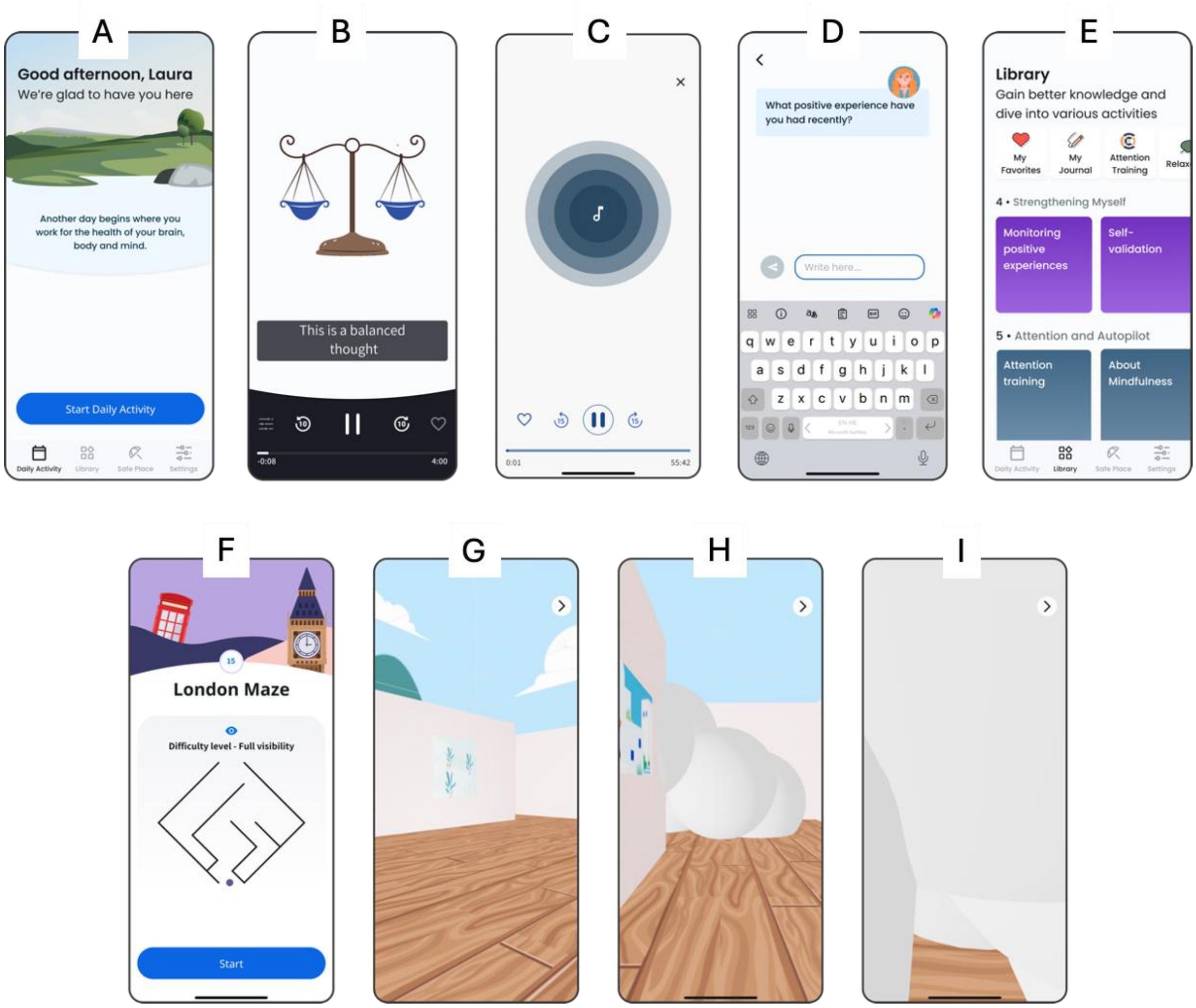

Supplementary Figure 5: Remepy-008 APP mobile application. Daily activity welcome screen (A), psychological interventions - video (B), psychological intervention – audio (C), psychological intervention – interactive activity (D), content library (E), top view map of HW maze used (F), full vision of the digital 3D egocentric navigation view (G), 50% random screening masked maze (H); blindfolded navigation maze (I).

4 References

1 Meunier, M., Saint-Marc, M. & Destrade, C. The Hebb-Williams test to assess recovery of learning after limbic lesions in mice. *Physiology & behavior* **37**, 909-913 (1986).

2 Aggius-Vella, E., Chebat, D.-R., Maidenbaum, S. & Amedi, A. Activation of human visual area V6 during egocentric navigation with and without visual experience. *Current Biology* **33**, 1211-1219. e1215 (2023).

3 Chebat, D.-R. *et al.* Sensory substitution and the neural correlates of navigation in blindness. *Mobility of visually impaired people: Fundamentals and ICT assistive technologies*, 167-200 (2018).

4 Huang, C.-C., Rolls, E. T., Feng, J. & Lin, C.-P. An extended Human Connectome Project multimodal parcellation atlas of the human cortex and subcortical areas. *Brain Structure and Function* **227**, 763-778 (2022).
